# A general approach to high-efficiency perovskite solar cells by any antisolvent

**DOI:** 10.1038/s41467-021-22049-8

**Published:** 2021-03-25

**Authors:** Alexander D. Taylor, Qing Sun, Katelyn P. Goetz, Qingzhi An, Tim Schramm, Yvonne Hofstetter, Maximillian Litterst, Fabian Paulus, Yana Vaynzof

**Affiliations:** 1 Kirchhoff Institute for Physics and Centre for Advanced Materials, Ruprecht-Karls-Universität Heidelberg, Heidelberg, Germany; 2 Integrated Center for Applied Physics and Photonic Materials, Technische Universität Dresden, Dresden, Germany; 3 Center for Advancing Electronics Dresden, Technische Universität Dresden, Dresden, Germany

**Keywords:** Solar cells, Semiconductors

## Abstract

Deposition of perovskite films by antisolvent engineering is a highly common method employed in perovskite photovoltaics research. Herein, we report on a general method that allows for the fabrication of highly efficient perovskite solar cells by any antisolvent via manipulation of the antisolvent application rate. Through detailed structural, compositional, and microstructural characterization of perovskite layers fabricated by 14 different antisolvents, we identify two key factors that influence the quality of the perovskite layer: the solubility of the organic precursors in the antisolvent and its miscibility with the host solvent(s) of the perovskite precursor solution, which combine to produce rate-dependent behavior during the antisolvent application step. Leveraging this, we produce devices with power conversion efficiencies (PCEs) that exceed 21% using a wide range of antisolvents. Moreover, we demonstrate that employing the optimal antisolvent application procedure allows for highly efficient solar cells to be fabricated from a broad range of precursor stoichiometries.

## Abstract (web summary)

Thin film deposition of perovskites by antisolvent engineering is commonly used, but the effect of processing parameters is not yet fully understood. Here, the authors identify two key factors that influence the film quality through a detailed structural and compositional study of perovskite layers fabricated by 14 different antisolvents.

## Introduction

Perovskites display a number of properties that directly translate to high performance in photovoltaic devices, such as low exciton binding energies^1^, long charge-carrier diffusion lengths^2^, and high absorption coefficients^3^. Such exceptional electronic behavior is tantalizing, and made more so by the low cost of their film fabrication^4^. Because they are made from earth-abundant materials and can be processed by low-temperature solution methods, perovskites have the potential to expand PV use by dramatically lowering the device payback time^5–7^. Researchers have repeatedly proven how to effectively combine these factors by simple spin-coating techniques, reporting power conversion efficiencies (PCEs) in excess of 20% and a current record of 25.5%^8^.

Among the various methods to deposit perovskite layers, such as spin-coating, inkjet printing^9,10^, thermal evaporation^11–13^, and many others, the most popular and commonly used is the so-called solvent-engineering method^14^. Here, the spin-coating of the perovskite precursor solution employs an antisolvent treatment to facilitate the removal of the host solvent(s) and initiate crystallization of the perovskite film. Several studies have lent insight into the optimal application of this step. For example, significant attention has been given to the effect that the time of the antisolvent dripping has on perovskite film formation. Through a variety of in and ex situ analysis techniques such as x-ray diffraction (XRD)^15–17^ and photoluminescence^18,19^, this research has revealed complex, composition-dependent liquid-crystal dynamics and competing crystallization routes that take place during the crucial moments of film formation. Antisolvent–solvent interactions (e.g., dipole–dipole) have been shown to modulate these dynamics^20^, and consequently, strong correlations are observed between antisolvent timing and the resultant film morphology, electronic quality, and photovoltaic performance, highlighting the paramount importance of the antisolvent application step in producing high-quality perovskite films and devices. Other parameters and techniques of antisolvent application have also been investigated, including antisolvent volume, temperature, dipping, additives, spin-coating parameters, and atmosphere^21–27^; however, a clear understanding of these variables is yet to emerge, and contradictory results and techniques are often reported. For example, while Ren et al.^28^ found that a cold antisolvent treatment resulted in superior devices, Taherianfard et al.^25^ found the opposite—that elevated temperatures produced the best results. One can find reported dripping delay times of anywhere between 5 and 30 s after spin-coating initiation, and optimal antisolvent volumes between 50 and 900 μL^29^. Even the choice of antisolvent is not straightforward^30–32^. Success has been demonstrated by researchers using antisolvents with no apparent commonality in physiochemical properties; both highly polar (such as ethyl acetate and isopropyl alcohol)^33–35^ and nonpolar solvents (toluene)^14^ have been used to form high-performance devices. For boiling point it is likewise; solvents with both extremely low (diethyl ether)^36^ as well as high (chlorobenzene)^37^ boiling points are commonly reported to yield high PCE devices. Even mixtures of antisolvents have been studied^38–42^. On the other hand, many solvents have been shown not to work well as antisolvents^21,32^. These widely varying reports and apparent discrepancies highlight the crucial need for a better understanding of the antisolvent deposition step.

In this work, we explore 14 antisolvents and demonstrate a general approach to achieving high-performance triple-cation Cs_0.05_(MA_0.17_FA_0.83_)_0.95_Pb(I_0.9_Br_0.1_)_3_ solar cells from any antisolvent. We show that by changing the duration of the antisolvent application, the device performance for certain antisolvents can be increased from nonfunctional to over 20% PCE. We show that antisolvents generally fall into three categories: those that favor short application times, those that are largely unaffected, and those that perform best with longer application times. By performing detailed morphological, compositional, and microstructural characterization of the perovskite layers, we identify the effects of the different classes of antisolvents on the perovskite film formation. We find that the solvent categorization is related to two fundamental properties: the degree of solubility of the organic iodides in the antisolvent and the miscibility of the antisolvent with the perovskite precursor host solvent(s). Depending on these two factors, tuning the application time results in efficient photovoltaic devices from any antisolvent. Finally, we also demonstrate that by using the optimal application time, it is possible to significantly expand the range of stoichiometries that lead to high device performance, thus eliminating the need for the commonly used PbI_2_ excess in the perovskite composition. Our results represent a crucial step toward a fundamental understanding of the role of antisolvents in perovskite film formation and demonstrate a general approach for efficient perovskite solar cells from any antisolvent.

## Results and discussion

### Adjusting the duration of antisolvent application

**Fig. 1:**
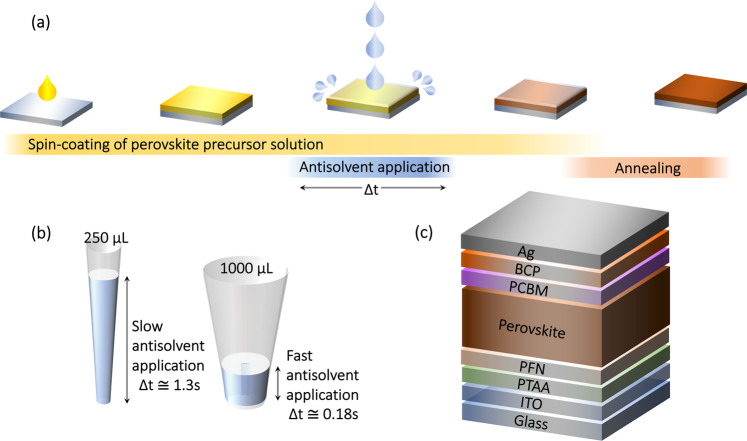
Deposition process and architecture of perovskite solar cells. **a** Schematic depiction of the perovskite layer fabrication process. **b** Illustration of the 1000 and 250 μL pipettes that were used to adjust the duration of the antisolvent application step. For the same applied force and solvent volume (200 μL), the extrusion rate is fast for the 1000 μL pipette and slow for the 250 μL pipette. **c** Schematic structure of the photovoltaic devices fabricated in this work.

The process of fabricating perovskite films by solvent engineering is schematically shown in Fig. 1a. In short, a concentrated perovskite precursor ink is deposited via spin-coating, followed by the application of an antisolvent at a fixed time before the end of the spin-coating procedure. The application of the antisolvent is not instantaneous and its duration (Δ*t*) has yet to be considered as an important factor for the perovskite film formation. To study this, a simple method to adjust the duration of antisolvent application is to employ two sizes of micropipettes, which dispense the same volume of solvent (200 µL) over different lengths of time. As is shown in Fig. 1b, the 1000 µL pipette has a significantly wider tip radius than the 250 µL, meaning the height of the solvent (for a given volume) in the tip is lower. Therefore, the plunger for the 1000 µL pipette must traverse a shorter distance to extrude the same volume of solvent as the 250 µL pipette, leading to a faster extrusion speed. To quantify these rates and the duration of antisolvent application, we filmed example antisolvent extrusions (“fast” and “slow”), and measured the time required for each via frame counting (Supplementary Note 1). This analysis resulted in approximate extrusion rates of ~1100 μL s^−1^ for fast (Δ*t* ≅ 0.18 s) and ~150 μL s^−1^ for slow (Δ*t* ≅ 1.3 s). Note that despite this technique, a certain variance between devices fabricated with the same intended speed is unavoidable simply due to human factors. However, as almost all perovskite PV devices are fabricated by hand, this human variance strengthens the comparability of our results to those of other research groups.

### The impact on solar cell performance

**Fig. 2:**
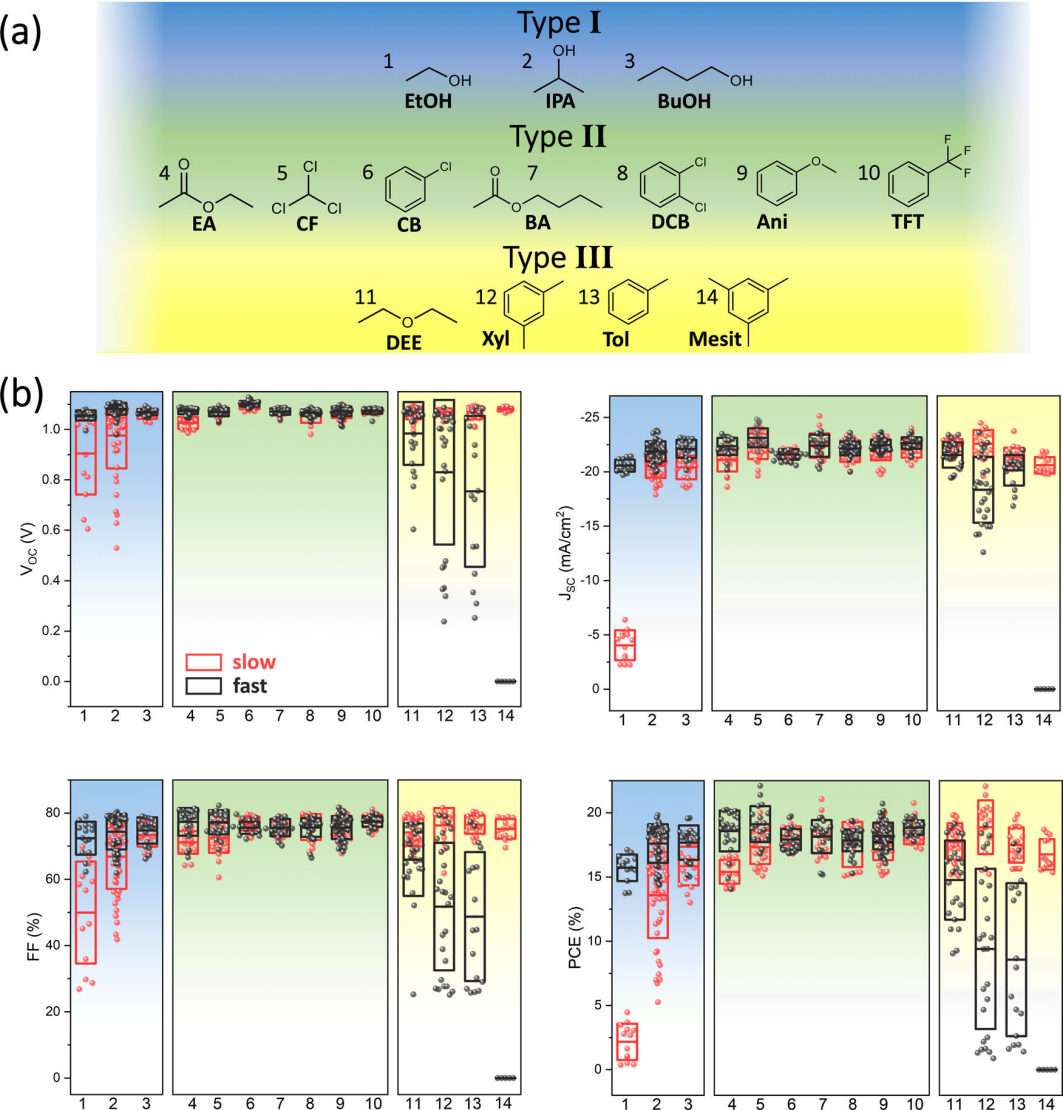
Perovskite PV performance as a function of antisolvent type and application rate. **a** The 14 antisolvents used in this experiment and **b** the photovoltaic performance of devices resulting from a fast or slow antisolvent application. The antisolvents are categorized as type I (blue), II (green), or III (yellow) according to their PV performance. The solvents are abbreviated as follows: 1: ethanol (EtOH), 2: isopropanol (IPA), 3: butyl alcohol (BuOH), 4: ethyl acetate (EA), 5: chloroform (CF), 6: chlorobenzene (CB), 7: butyl acetate (BA), 8: 1,2-dichlorobenzene (DCB), 9: anisole (Ani), 10: trifluorotoluene (TFT), 11: diethyl ether (DEE), 12: m-xylene (Xyl), 13: toluene (Tol), are 14: mesitylene (Mesit). The center line denotes mean value, box limits are upper and lower quartiles.

To investigate the effect of adjusting the duration of the antisolvent application step, we fabricated nearly 800 triple-cation Cs_0.05_(MA_0.17_FA_0.83_)_0.95_Pb(I_0.9_Br_0.1_)_3_ perovskite PV devices across 14 different antisolvents (see Supplementary Table 1 for antisolvent properties, and Fig. 1c for full device stack used), and compared their photovoltaic performance when using a fast or slow antisolvent application (called fast and slow devices for simplicity). The chemical structures of the antisolvents are depicted in Fig. 2a, with the solvents categorized according to their photovoltaic performance. A summary of the resulting open-circuit voltage (*V*_OC_), short-circuit current (*J*_SC_), fill factor (FF), and PCE is shown in Fig. 2b. Broadly speaking, almost every antisolvent yields devices with PCEs approaching or exceeding 20%, with the best performers having a consistent *V*_OC_ of ~1.1 V, a FF between 75 and 83%, and a *J*_SC_ of 22–23 mA cm^−2^. When considering fast versus slow antisolvent application, differences between the solvents are immediately apparent. The antisolvents can be placed into three categories, types I–III, based on these differences. Type I antisolvents, consisting of the alcohol series ethyl, isopropyl, and butyl alcohols, result in better devices when the antisolvent is applied quickly. As shown in Fig. 2b, fast application of the antisolvent leads to an equally high *V*_OC_ and similar values for the *J*_SC_, FF, and PCE, while using a slow application negatively affects the performance, from a small, but noticeable, difference for butanol to near nonfunctionality (<5% PCE) for ethanol. Furthermore, especially in the FF, a significantly broader distribution of values is obtained for the slow devices. The type III antisolvents, on the other hand, show the exact opposite trend. Here, slow antisolvent application yields good performance with a narrow distribution, while the fast devices perform between slightly and significantly worse. Mesitylene has the most extreme difference: while slow application yields competitive performances, fast extrusion does not result in a single functional device. Concurrently, the fast devices of the other antisolvents in this category contain a higher proportion of short-circuited devices. In the final category, the performances of type II antisolvents are largely unaffected by the duration of antisolvent application. Notably, for all the tested antisolvents, the highest performance devices are at roughly the same level, reaching average PCEs around 18% and champion pixels of over 21%. Example J–V curves for fast and slow pixels are shown in Supplementary Fig. 1.

To examine the boundary of antisolvent application rate which can be categorized as slow or fast, we fabricated a series of devices using a representative antisolvent from each type in which the application rate was gradually varied (Supplementary Fig. 2). We find that, for the antisolvents used, the rate of application that leads to higher performances is ~1100–1500 µL s^−1^ for IPA, while the boundary for slow application for mesitylene is ~100–150 µL s^−1^.

### Type I antisolvents

**Fig. 3:**
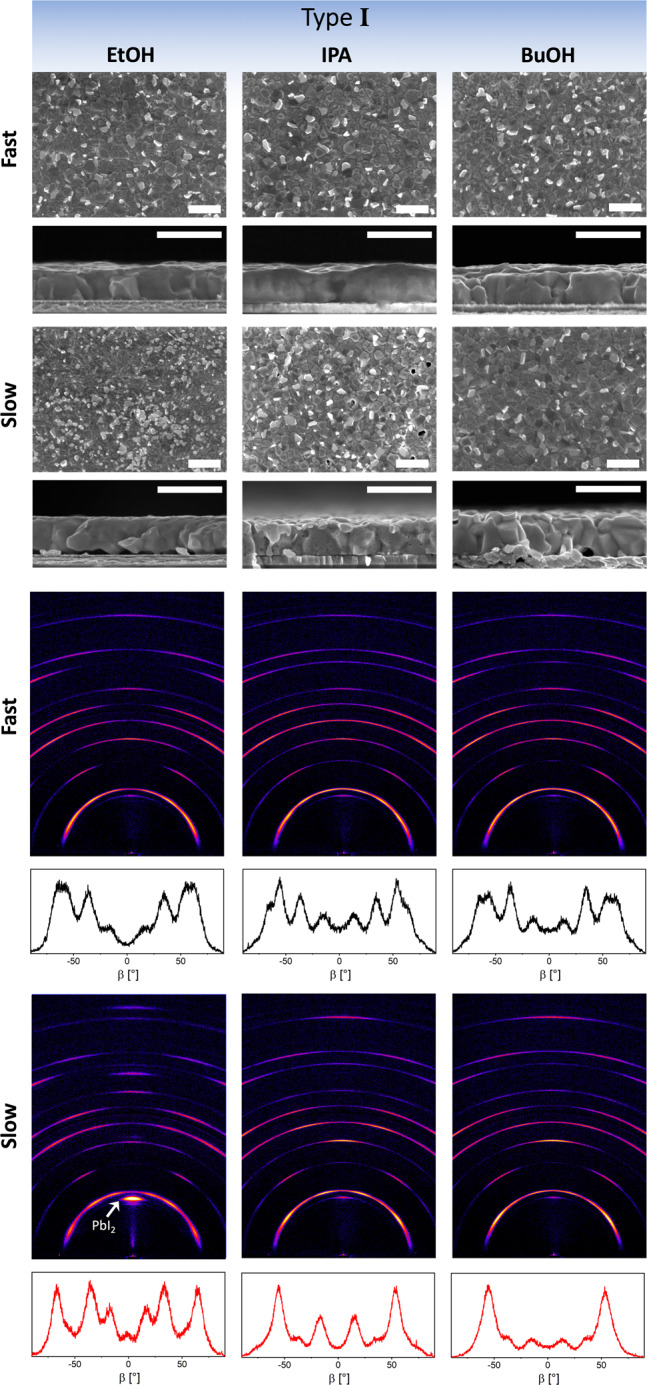
Microstructural and structural characterization of perovskite films deposited by type I antisolvents. Top: surface and cross-sectional scanning electron microscopy images of perovskite films formed from type I antisolvents (EtOH, IPA, and BuOH). Scale bar is 1 μm. Bottom: 2D XRD maps and corresponding ß integration of the (100) reflection to visualize changes in grain orientation. The arrow highlights the reflection associated with PbI_2_.

**Fig. 4:**
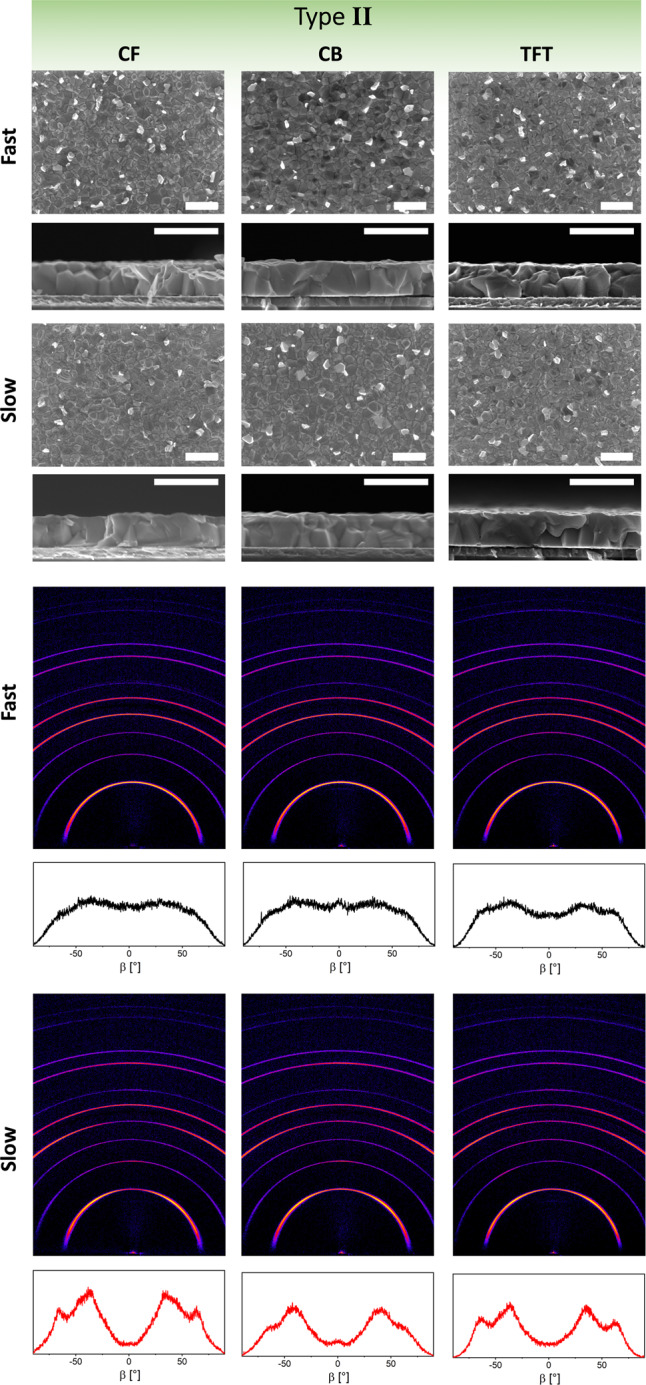
Microstructural and structural characterization of perovskite films deposited by type II antisolvents. Top: surface and cross-sectional scanning electron microscopy images of perovskite films formed from selected type II antisolvents (CF, CB, and TFT). Scale bar is 1 μm. Bottom: 2D XRD maps and corresponding ß integration of the (100) reflection to visualize changes in grain orientation.

**Fig. 5:**
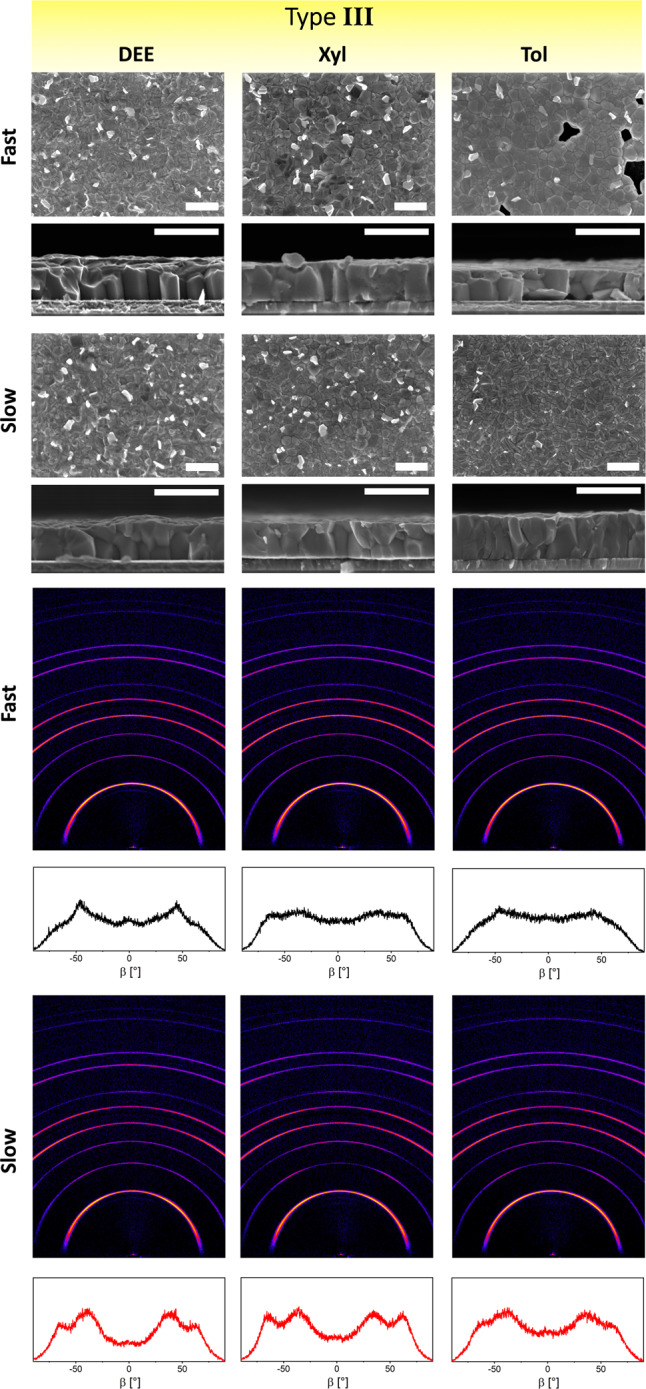
Microstructural and structural characterization of perovskite films deposited by type III antisolvents. Top: surface and cross-sectional scanning electron microscopy images of perovskite films formed from selected type III antisolvents (DEE, Xyl, and Tol). Scale bar is 1 μm. Bottom: 2D XRD maps and corresponding ß integration of the (100) reflection to visualize changes in grain orientation.

Microstructural characterization of films fabricated from type I antisolvents reveals stark differences between slow and fast antisolvent application (Fig. 3). In the case of fast antisolvent application, scanning electron microscopy (SEM) images show dense and compact perovskite films with some phase-separated lead iodide (bright features in the SEM, Figs. 3–5) on the surface^43^. Cross-sectional SEM confirms that the films consist of relatively large perovskite grains, which extend throughout the entire film thickness. In contrast, films formed by slow antisolvent application exhibit a far inferior microstructure. The surfaces of these films contain significantly higher amounts of lead iodide, particularly evident in the EtOH and IPA samples, and often display pinholes or small voids. In addition, cross-sectional imaging reveals the formation of large voids at the interface with the hole-extraction layer PTAA. The apparent film quality observed via SEM aligns well with the PV results; as an example, the large density of voids observed in the slow EtOH films leads to a very poor hole extraction, greatly limiting the *J*_SC_ and the overall photovoltaic performance. The large distribution of the photovoltaic performance of the slow IPA films is likely to be caused by the formation of pinholes and small voids in the devices’ active layers. Butanol, on the other hand, shows the fewest voids at the interface with poly(triaryl amine) (PTAA) for the slow antisolvent application, consistent with the smallest difference in PV performance.

Structural characterization by 2D XRD reveals another interesting feature of films formed by type I antisolvents, as shown in Fig. 3. While the films exhibit the characteristic perovskite diffraction patterns with a cubic unit cell with lattice constants a = b = c = 6.305 Å^44^, the distribution of intensities along the Debye rings (as can also be seen in the ß integration) demonstrates that type I antisolvents result in polycrystalline perovskite films with a remarkably high degree of preferred orientation. A fast antisolvent application lowers this degree of ordering, but it is still significantly higher than that of other antisolvents and previous literature reports^45^. In addition, the XRD measurements from all films formed by type I antisolvents show a significant contribution of lead iodide (marked with an arrow in Fig. 3), with EtOH displaying the largest signal, then IPA, and BuOH the smallest. Furthermore, the PbI_2_ signal is amplified in the case of slow antisolvent application versus fast. This is in agreement with the observations by SEM and x-ray photoemission spectroscopy (Supplementary Fig. 3).

### Type II antisolvents

From the seven type II antisolvents, Fig. 4 shows the structural and microstructural characterization of representative three antisolvents: CF, CB, and TFT, with the other four shown in Supplementary Fig. 4. SEM imaging reveals that all type II antisolvents result in the formation of high-quality, uniform, and pinhole-free films, independent of the duration of antisolvent application. The films show a far smaller lead iodide content than type II, evident both in the surface SEM images and the 2D XRD maps. X-ray spectroscopy (XPS) measurements confirm the very similar surface composition of all films formed by fast and slow application of all type II antisolvents.

Similar to the type I antisolvents, the films exhibit perovskite diffraction patterns with the same cubic unit cell parameters, but unlike type I, no significant preferential orientation is observed for films formed with type II antisolvents, in particular those formed via a fast application. The minor differences in the degree of orientation between the antisolvent application times do not seem to correspond with differences in the photovoltaic performance of the devices.

### Type III antisolvents

The differences between films formed by slow and fast application of type III antisolvents are easily visible by eye (Supplementary Fig. 5). Films deposited via a fast antisolvent application result in only a portion of the film—corresponding to the area where the antisolvent is dispensed—appearing dark and shiny, apparently indicating the formation of the desired perovskite phase. Other areas, however, take on a hazy appearance or do not change to the black perovskite phase at all. This is made apparent by optical transmission microscopy: here, the sample shows a clear boundary between the area where the black perovskite phase forms and the area where it remains unconverted (Supplementary Fig. 6). Interestingly, SEM imaging reveals that this central perovskite region appears similar to the other high-performing films (Fig. 5 and Supplementary Fig. 7), apart from toluene which exhibits a significant number of pinholes. Nevertheless, 2D XRD confirms that the central region is a polycrystalline perovskite with no significant preferential orientation. It is likely that this partial conversion is a result of the central spot, where the antisolvent is dispensed, being exposed to the antisolvent for a longer amount of time than the surrounding areas, thus nearing the conditions of the slow application. The varying degrees of overlap of this central perovskite area with the device’s electrodes lead to a large distribution of photovoltaic performance with significant losses due to shunts via the unconverted areas.

In contrast, slow application of type III antisolvents results in compact and uniform films with a smaller overall amount of residual PbI_2_ (Fig. 5). These films are very similar in microstructure and crystalline orientation to those formed by type II antisolvents, with many devices showing photovoltaic performance with efficiencies surpassing 20%. This is especially noteworthy in the case of mesitylene, where fast application of mesitylene leads to particularly poor films, resulting in no functional photovoltaic devices.

### Origin of antisolvent categorization

**Fig. 6:**
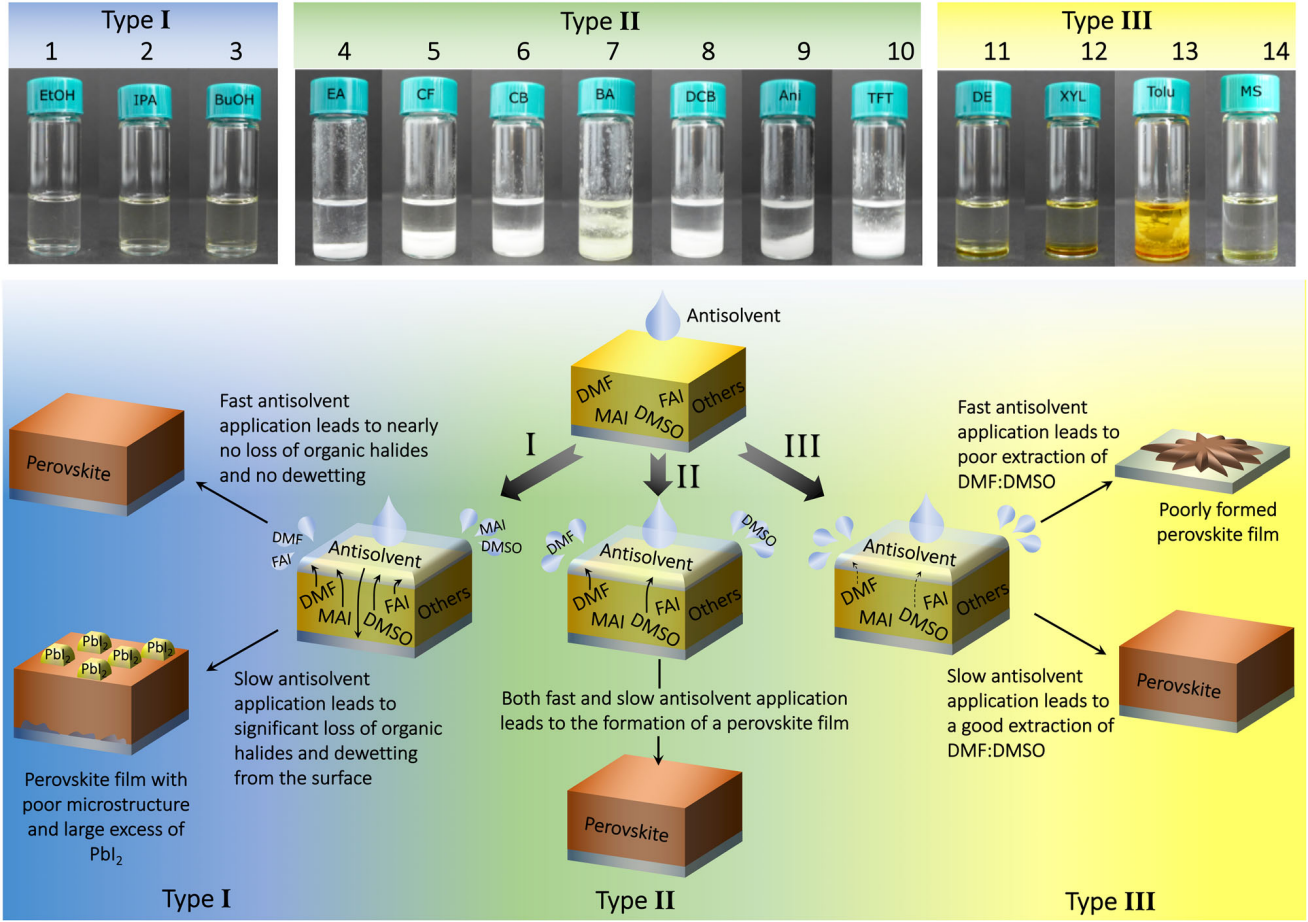
Summary of the processes taking place during perovskite film formation for the three antisolvent types. Top: solubility of MAI in a solution of DMF:DMSO:antisolvent, meant to simulate the perovskite film intermediate phase during the antisolvent step of fabrication. Bottom: summary of the various mechanisms involved in perovskite film formation by the different categories of antisolvents.

The significantly damaged microstructures of slow type I and fast type III, in conjunction with altered ratios of phase-separated PbI_2,_ suggest that the relative solubility of the precursor components in the various antisolvents plays a key role in film formation, and that by changing the duration of the antisolvent application speed, one can tune this effect^46^. To explore this, we prepared 2 M methylammonium iodide (MAI) or formamidinium iodide (FAI) solutions in 4:1 dimethylformamide:dimethyl sulfoxide (DMF:DMSO), and tested the solubility/miscibility in each antisolvent. The volume ratio was chosen to be 6:1 antisolvent:host, as this is approximately the volume ratio used in the perovskite fabrication process. The results of this test for MAI are shown in Fig. 6, and for FAI are shown in Supplementary Fig. 8a.

While the results for FAI are less clear, for MAI a strong distinction between the three types of antisolvents can be seen. Type I antisolvents result in a well-mixed and clear solution, while for type II, the solvents are well mixed but a significant amount of white precipitates are present. For type III, a liquid phase separation in combination with a yellowish color change is observed (see Supplementary Note 11 for more details).

Armed with these results, we can understand the mechanisms leading to the trends discussed above. In a simplistic model, the application of the antisolvent triggers two distinct processes. Adding the liquid antisolvent on top of the thinned precursor solution extracts the DMF:DMSO from the underlying layer via diffusion. During this period, the constant supply of neat antisolvent over the precursor layer maintains a high gradient for diffusion and makes this process very efficient. The extraction of the solvent from the precursor solution simultaneously triggers nucleation and solidification of the perovskite material, resulting in polycrystalline films as observed. However, precursor molecules may also diffuse into the antisolvent layer, in addition to the solvent molecules. The relative differences in the interactions between solvent and precursor material with the given antisolvent determine the effectiveness of the antisolvent treatment at removing the DMF:DMSO while preserving the perovskite composition.

For type I antisolvents, we observe increasing solubility of the organic precursors as the alkyl chain length of the alcohol decreases (i.e., the solubility is highest in EtOH and lowest in BuOH), as well as an overall high miscibility with the DMF:DMSO solvent mixture (Supplementary Fig. 8b, c). Concurrently, films formed by this type of antisolvent possess a large amount of residual PbI_2_ when compared to films formed by the other antisolvent types. This suggests that, along with the DMF:DMSO, a considerable amount of the organic halides is also removed by these antisolvents, damaging the film microstructure and leaving behind PbI_2_ which cannot convert to perovskite, as the stoichiometry has been irrevocably altered. This explains why the performance difference between fast and slow is the largest for EtOH, and decreases as the solubility of the organic precursors in the antisolvent is reduced. This is consistent with the use of methanol (MeOH) as the antisolvent: the solubility of the organic precursors in MeOH is so high that they are completely removed from the film during the antisolvent application, yielding only a yellow film of residual PbI_2_ (Supplementary Fig. 9). In other words, the type I antisolvents partially act as a regular solvent for the organic components, and as an antisolvent only toward the inorganic components. However, due to the differences in diffusion rates, short application times are ineffective at removing the organic halides, but are still sufficient to remove the DMF:DMSO, thus yielding high-quality films with a good PV performance.

The type II antisolvents have the ideal combination of properties. They exhibit low solubility for the organics, as indicated by the amount of precipitates seen in Fig. 6, but are still miscible with the DMF:DMSO host solvent and therefore provide effective removal of the solvent mixture. Due to the large mismatch in diffusion rates for these antisolvents, the duration of the antisolvent application is largely irrelevant, as they will only act to remove the DMF:DMSO while leaving the perovskite composition intact. However, as shown in Fig. 2, certain type II antisolvents still display a difference between fast and slow performance. This is caused by their low, but nonnegligible, solubility for the organic iodides—EA, for example, possesses the smallest amount of precipitates of all the type II antisolvents, and also has the largest performance difference, indicating that it sits somewhere between type I and II. This is the reason for EA’s inclusion in type II: despite having similar PV behavior to BuOH, the solubility test shows a clear distinction between the two.

As noted previously, the type III antisolvents often possess poor film coverage when formed via a fast antisolvent application. This is likely caused by the immiscibility of the solvents, indicated by the liquid phase separation observed in the top panel of Fig. 6. When applied quickly, there is inadequate time for the DMF:DMSO to diffuse across the liquid–liquid interface into the antisolvent, and the film coverage suffers as a result, analogous to film fabrication without any antisolvent. Only in the very center, where the film is most exposed to the antisolvent, is the host solvent effectively removed and a perovskite crystal phase formed. Prolonging contact between the antisolvent and precursor layers allows the relatively slow diffusion process enough time to proceed to completion. Indeed, films fabricated via a slow antisolvent application, which is sufficient to extract the host DMF:DMSO solvents, result in high-quality perovskite films with excellent photovoltaic performance. Figure 6 summarizes the mechanisms involved in perovskite film formation by the different categories of antisolvents.

Investigation of other processing parameters of antisolvent application supports these observations. For example, changing fabrication parameters that do not influence the solubility of organic precursors in the antisolvent, or its miscibility with the host solvents, should not have any effect on the resulting device performance. To confirm this, we fabricated devices while varying the distance between the pipette and the substrate from 0 to 4 cm, and observe no change in the photovoltaic performance (see Supplementary Fig. 11). Next, we tested the fabrication environment by fabricating photovoltaic devices using six different antisolvents (two from each type) in an N_2_-filled glovebox instead of a dry air glovebox. Again, we observe that the change of environment does not influence the difference between fast and slow application, with the categorization of antisolvents and the resultant photovoltaic performance remaining unchanged when compared to those of devices fabricated in dry air (see Supplementary Fig. 12). On the other hand, changing a processing parameter that influences the solubility of the organic precursors in the antisolvent should lead to a change in the photovoltaic performance. It has been recently suggested that increasing the temperature of the antisolvent may lead to an increase of devices performance^25^. To explore this, we fabricated photovoltaic devices using different antisolvents, but at elevated temperatures of 35 and 50 °C. This increase in antisolvent temperature may lead to a change in the solubility of the organic precursors in the antisolvent, or may influence the process of perovskite crystallization. Indeed, a comparison of the performance of the heated antisolvent devices to those made at room temperature (Supplementary Fig. 13) reveals that elevating the antisolvent temperature affects the PV performance. While in the case of CB a marginal improvement is observed for 35 °C, in agreement with literature^25^, other type II antisolvents such as TFT and anisole display a different behavior. Upon increasing the antisolvent temperature, they begin to behave more like type I, i.e., their slow deposition is detrimental to the device performance, caused by the increased solubility of organic precursors in the heated antisolvent. The examined type I and type III antisolvents also show a decrease in device performance upon increasing the antisolvent temperature. Finally, we examined the effect of varying the volume of antisolvent (Supplementary Fig. 14). It is important to note that this parameter indirectly influences the rate of antisolvent application, as it takes longer to dispense a larger volume of antisolvent from the pipette. Indeed, increasing the volume of a representative type I antisolvent (EtOH) leads to a decrease in photovoltaic performance, as the longer application due to the larger volume is unfavorable for type I antisolvents. On the other hand, increasing the volume of toluene, a type III antisolvent, led to a continuous increase in PCE, as slower application is beneficial for this type of solvents. We stress that throughout our study, we kept the volume of antisolvent fixed (200 μL), ensuring that the antisolvent application rate was kept as consistent as possible.

To investigate whether the rate of antisolvent application influences the stability of the fabricated perovskite layers, we monitored their absorption over a period of 4 weeks (Supplementary Fig. 15) and monitored the performance of devices from one representative antisolvent from each type over a period of 20 days (Supplementary Fig. 16). Both the films and devices were unencapsulated and were stored in ambient air in the dark in between measurements. We observe no significant differences in the stability of the perovskite layers and devices, suggesting that once a perovskite layer is formed, the choice of antisolvent no longer plays a role in the film degradation dynamics.

The development of large-scale fabrication procedures of perovskite photovoltaic devices is critically important for their future integration into industrial applications^47,48^. While making large-area devices is beyond the scope of the current study, we tested whether varying the rate of antisolvent application can be used as a method to fabricate high-quality perovskite layers on large substrates. For this purpose, we fabricated perovskite films on 5 × 5 cm^2^ samples from representative antisolvents from each type (Supplementary Fig. 17). We observe very similar results to those described above, namely that slow type I application and fast type III antisolvent application lead to poor film quality. These results highlight that controlling the rate of antisolvent application is also an important parameter for large-area fabrication of perovskite devices.

Recently, significant efforts have been devoted to investigate MA-free perovskite compositions with impressive device performance demonstrated by a range of methods^49–51^. We note that while the focus of this work was photovoltaic devices with a triple-cation perovskite composition, we observe that tuning the duration of antisolvent application is similarly critical in MA-free devices (Supplementary Fig. 18), owing to the presence of FAI in these devices and its similar behavior to what has been discussed here. While the assignment of each antisolvent into a particular category might differ from that described here for the MA containing perovskite compositions, these observations confirm the broader applicability of our approach for other perovskite solar cells fabricated by the solvent-engineering method.

### Role of precursor solution stoichiometry

Various groups have reported an increase in performance and reproducibility when an excess of PbI_2_ was included in the precursor solution^52,53^. This partially stems from the fact that the vast majority of research groups treat Cs as an additive rather than a component in their stoichiometry calculations, i.e., setting the ratio of (FA + MA):Pb equal to one. In our case, we include Cs as a component and thus (Cs + MA + FA):Pb = 1; this difference in methodology results in what others would consider a 5% lead excess. For reference, Saliba et al.^54^ reported best results with an 8% excess (as calculated without considering Cesium). While some reports suggest that reducing any residual PbI_2_ is indicative of a superior performing film^54–56^, other reports have found their highest performing devices contain more PbI_2_ than the controls, leaving the role of PbI_2_ unresolved^49,57–62^. It is noteworthy that excess PbI_2_ has been shown to lead to a reduction in device stability^63,64^. Because the results of our examination of the effect of antisolvent extrusion rate in this study revealed alterations to the stoichiometry, microstructure, and residual PbI_2_ in our triple-cation devices, we also examined how the fast versus slow antisolvent application interacts with variations in the initial precursor stoichiometry.

To investigate how changing the duration of the antisolvent application step interacts with variations in the precursor solution stoichiometry, we measured the performance of photovoltaic devices fabricated with a varying initial amount of organic precursors, i.e., Cs_0.05_(FA_0.83_MA_0.17_)_0.95*·X*_Pb(I_0.9_Br_0.1_)_3_ with *x* ranging from 0.9 to 1.1. We selected anisole, which as a type II antisolvent reliably produces high-performance devices with very little difference between fast and slow antisolvent application.

**Fig. 7:**
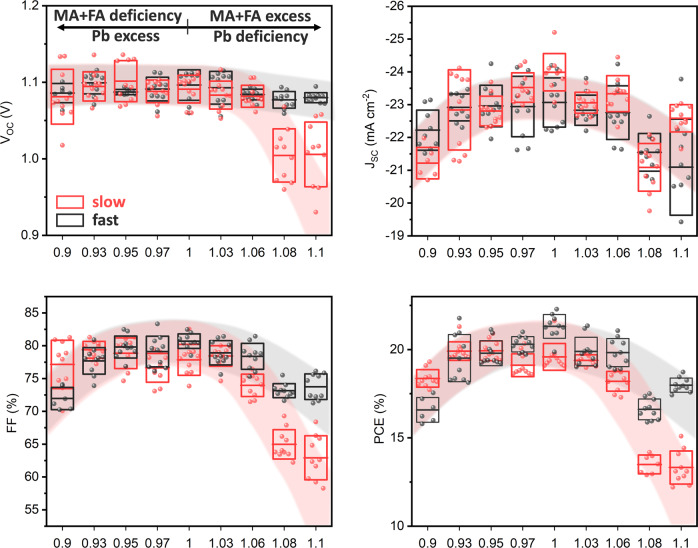
PV performance of triple-cation perovskite solar cells with varying stoichiometric ratios, for fast and slow antisolvent application rates. [MA + FA]_1_ is the amount of organics in an exactly stoichiometric precursor solution. For clarity, only the top ten devices for each category are shown. The center line denotes mean value, box limits are upper and lower quartiles. The red and black shaded regions are shown as a guide for the eye.

Figure 7 shows that for both antisolvent applications, the PCE remains largely unaffected by the deliberate introduction of a deficiency of organic precursors, with only the films that are 10% deficient in organic iodides (*x* = 0.9) resulting in a lower performance. On the other hand, excess of organic iodides leads to a significant difference in the performance of devices fabricated via the slow and fast antisolvent application. In the case of a fast application, the devices maintain their performance up to 6% excess, and show only a moderate reduction in performance for larger *x* values. Contrary to this, slow application causes a loss of performance already at 3% excess of organic iodides and results in a severe loss of efficiency for higher excess values. Note that, for easier visualization due to a significant amount of overlapping data, Fig. 7 only displays the top ten devices for each category. However, the trend is identical for all devices, and for the interested reader, a full display of all devices’ performance is shown in Supplementary Fig. 19.

These results suggest that the common use of excess lead iodide (i.e., deficiency in organic cations) does not necessarily originate solely from an innate advantage in terms of device efficiency, as equally efficient devices can be fabricated with a similar excess of organic cations, provided the right duration of the antisolvent application is adopted. Instead, it is likely that the use of lead iodide excess is related to the enhanced reproducibility of the performance of solar cells fabricated by different researchers (with naturally differing rates of antisolvent application) in this stoichiometry regime. Importantly, we observe that the highest performance is achieved for fully stoichiometric devices (*x* = 1, PCE = 22.4%), thus eliminating the need to introduce excess PbI_2_ into the precursor solution.

In summary, we present a simple approach to the fabrication of high-efficiency perovskite photovoltaic devices from any antisolvent. We demonstrate that antisolvents can be categorized in three groups depending on two factors: (1) their ability to dissolve the organic precursor components and (2) their miscibility with the host perovskite solution solvents. These two factors dictate the optimal application rate for each antisolvent, allowing the formation of high-quality perovskite layers and efficient photovoltaic devices from any antisolvent. Moreover, we demonstrate that by employing this optimal antisolvent application time, high-efficiency devices can be made from a broad range of precursor stoichiometries, tolerating both excess and deficiency of organic iodides by up to 6%. These results not only enhance the fundamental understanding of the role of antisolvents in the film formation of perovskite solar cells but also provide a simple route to achieve high-efficiency devices with increased reproducibility.

## Methods

### Materials

Glass substrates, precoated with a central ITO stripe, were purchased from Psiotec Ltd. Large-area ITO coated glass substrates were acquired from MSE supplies. MAI (CH_3_NH_3_I) and FAI (HC(NH_2_)_2_) were purchased from GreatCell Solar. PbI_2_ and PbBr_2_ were purchased from TCI. PC_60_BM was purchased from Solenne BV. All other materials and solvents were purchased from Sigma-Aldrich. All materials were stored in a nitrogen-filled glovebox and used as received.

### Solution preparation

Perovskite films were fabricated from precursor solutions using the recipe Cs_0.05_(FA_0.87_MA_0.13_)_0.95_Pb(I_0.9_Br_0.1_)_3_. We used a sequential solution method to prepare exactly stoichiometric 1 M precursor solutions. To do so, 2 M solutions of CsI, PbI_2_, and PbBr_2_ were prepared by dissolving each in a 4:1 volume ratio (v/v) mixture of DMF:DMSO and heating at 180 °C, and CsI in pure DMSO at 150 °C. After being dissolved, the volume of these solutions expands due to the presence of the solute, thus their true concentration will be lower than what is found by simply dividing the molecular weight (M_w_) of the powder by the volume of the solvent added. To determine this true concentration, the mass of a known volume of each solution was measured, from which the molarity can be calculated. Once the true concentration was known, each solution was diluted by adding the appropriate solvent until the desired concentration of 1.155 M was reached, and then the CsI, PbI_2_, and PbBr_2_ solutions were mixed in a volume ratio of 0.05:0.85:0.15, yielding a 1.1 M solution of Cs_0.05_Pb(I_1.75_Br_0.3_), which we term the inorganic stock solution. In two separate vials FAI and MAI powders were added and weighed, into which the appropriate amount (0.95:1 molar ratio) of inorganic stock was added. This creates two new solutions, of the formula Cs_0.05_(FA or MA)_0.95_Pb(I_0.9_Br_0.1_)_3_. Finally, these two solutions were mixed in a 5:1 v/v ratio, in order to achieve the final molecular formula Cs_0.05_(FA_0.83_MA_0.17_)_0.95_Pb(I_0.9_Br_0.1_)_3_. For the MA-free devices, the last step was omitted to yield a precursor solution of Cs_0.05_(FA)_0.95_Pb(I_0.9_Br_0.1_)_3_.

### Device fabrication

PV devices were fabricated in the device stack glass/ITO/PTAA/PFN-Br/CsFAMA/PCBM/BCP/Ag. First, Glass/ITO substrates were sequentially cleaned by sonication in 2% Hellmanex detergent, deionized water, acetone, and isopropyl alcohol. After being blown dry, the substrates were exposed to an oxygen plasma at 100 mW for 10 min to remove any residual contamination. Immediately after plasma cleaning, the devices were transferred to a drybox (<2% relative humidity), where PTAA was spin-coated from a 1.5 mg mL^−1^ solution in anhydrous toluene at 2000 RPM for 30 s, followed by a 10 min annealing step at 100 °C. After letting the substrates cool for 5 min, a thin layer of PFN-Br was then spin-coated from a 0.5 mg mL^−1^ solution in anhydrous methanol at 5000 RPM for 30 s, with no thermal annealing. This layer is required to increase the wettability on the PTAA film. The perovskite layer was spin-coated with a two-step recipe, first at 1000 RPM for 10 s followed by 5000 RPM for 30 s. Two hundred microliters of anhydrous antisolvent were dripped onto the substrate 5 s as a continuous droplet before the end of the second step, at either a fast or a slow rate. The distance between the pipette tip and substrate was ~5 mm. The as-spun samples were annealed at 100 °C for 30 min and then transferred to a nitrogen-filled glovebox.

For the electron transport side, phenyl-C61-butyric acid methyl ester (PCBM) was spin coated dynamically from a 20 mg mL^−1^ solution in anhydrous chlorobenzene at 2000 RPM for 30 s, followed by a 10 min anneal at 100 °C. After letting the substrates cool for 5 min, bathocuproine (BCP) was spin-coated dynamically from a 0.5 mg mL^−1^ solution in anhydrous IPA at 4000 RPM for 30 s, with a 5 min anneal at 70 °C. To complete the devices, the samples were then transferred, without breaking the inert atmosphere, to a thermal evaporator where 80 nm silver electrodes were deposited at an initial rate of 0.01 nm s^−1^ for the first 15 nm, then 0.1 nm s^−1^ for the remainder.

### Large-area film fabrication

The deposition procedure for 5 × 5 cm^2^ large-area substrates was identical to that from the small-area substrates, however 1000 µL of antisolvent was employed instead. Images depicting the film quality were taken using a standard smartphone camera. The authors affirm that human research participants provided informed consent for publication of the images in Supplementary Fig. 17.

### Adjusting the precursor stoichiometry

During the precursor solution preparation, an inorganic stock solution was created consisting of Cs_0.05_Pb(I_1.75_Br_0.3_), which was then added to MAI and FAI powders to create the final perovskite solution Cs_0.05_(FA_0.83_MA_0.17_)_0.95_Pb(I_2.7_Br_0.3_). To adjust the [MAI + FAI]:Pb ratio, a smaller volume of inorganic stock solution was added instead of the exact amount, creating an (organic) overstoichiometric perovskite solution of a known volume. This solution was then used as above to fabricate a number of devices, and the resulting solution volume calculated. Then, to incrementally adjust the stoichiometry, a small volume of the inorganic stock was added to the precursor solution to adjust the stoichiometry to the desired ratio. This process was repeated until the full stoichiometry range was covered.

### J–V characterization

Current density–voltage measurements were performed in ambient conditions under simulated AM 1.5 light with an intensity of 100 mW cm^−2^ (Abet Sun 3000 Class AAA Solar Simulator). The intensity was calibrated using a Si reference cell (NIST traceable, VLSI), and corrected by measuring the spectral mismatch between the solar spectrum, reference cell, and the spectral response of the PV device. The mismatch factor obtained was ~1.1. Cells were scanned using a Keithley 2450 source measure unit from 1.2 to 0 V and back, with a step size of 0.025 V and a dwell time of 0.1 s, after light soaking for 2 s at 1.2 V. The pixel area was 3 mm × 1.5 mm.

### X-ray photoemission spectroscopy

Samples for XPS (glass/ITO/PTAA/PFN-Br/CsFAMA) were prepared as described above and transferred into the ultrahigh vacuum chamber of the XPS system (Thermo Scientific ESCALAB 250Xi, Specs PHOIBOS 100) for measurement. All measurements were performed in the dark, and five spots per sample were measured and averaged to acquire the statistics. XPS measurements were performed using an XR6 monochromated AlKα source (hv = 1486.6 eV) and a pass energy of 20 eV.

### 2D XRD

2D XRD measurements of the CsFAMA films on glass/ITO/PTAA/PFN-Br were conducted in ambient air at room temperature using a Rigaku SmartLab diffractometer with a 9 kW rotating copper anode and a 0.2 mmφ collimator. The 2D diffraction maps were recorded in a coupled θ/2θ scan from 0° to 55°, 2° min^−1^ with a HyPix3000 detector (detector distance of 110 mm) utilizing a knife edge to lower the background scattering and a beam blanker to block the direct beam. All 2D diffraction patterns were background corrected by subtracting a measurement of a plain glass/ITO/PTAA/PFN-Br sample. The corresponding and indexed 1D profiles as a result of an integration of a central area (azimuth angle *β* = ±10°) for all films can be found in the Supplementary Fig. 20. To visualize the differences in preferred orientation, the first perovskite reflection (100) was integrated from 2*θ* = 13–15° and azimuth angle *β* = ±90°.

### Scanning electron microscopy

SEM was performed using a JSM‐7610F FEG‐SEM (Jeol). Samples were mounted on standard SEM holders, grounded via the patterned ITO stripe using conductive Ag paste to avoid sample charging and placed inside the analysis chamber at a vacuum pressure < 10^−6^ mbar. Top-view images were recorded using an acceleration voltage of 1.5 kV and ×20,000 magnification. Cross-section images of freshly cleaved samples at 90° were obtained with acceleration voltages of 5 kV and magnifications of ×40,000. All images utilized the SEI detector (in-lens) above the sample, collecting a combination of secondary electrons and backscattered electrons, to visualize differences in surface and material properties. To confirm that the bright areas observed in the top-view images correspond to PbI_2_ (a material of higher density) and are not simply charged grains, the same perovskite area was also imaged using a low-angle backscatter electron detector (see Supplementary Fig. 21).

### Reporting summary

Further information on research design is available in the Nature Research Reporting Summary linked to this article.

## Supplementary information

Supplementary Information

Solar Cells Reporting Summary

## Data Availability

The data that support the findings of this study are available on reasonable request from the corresponding author.
