# Supplementary material for: A general approach to high-efficiency perovskite solar cells by any antisolvent
Source: Nat Commun. 2021 Mar 25;12:1878. doi: 10.1038/s41467-021-22049-8 (PMC7994557; doi:10.1038/s41467-021-22049-8)
Supplement: Supplementary file 1 — Supplementary Information [file 41467_2021_22049_MOESM1_ESM.pdf]

Supporting Information

A General Approach to High Efficiency Perovskite Solar Cells by Any Antisolvent

*Alexander D. Taylor,[1,2,3] Qing Sun,[1] Katelyn P. Goetz,[1,2,3] Qingzhi An,[1,2,3] Tim Schramm,[2] Yvonne Hofstetter,[1,2,3] Maximillian Litterst,[1] Fabian Paulus,[1,3] and Yana Vaynzof[1,2,3]**

[1] Kirchhoff Institute for Physics and Centre for Advanced Materials, Ruprecht-Karls-Universität Heidelberg, Im Neuenheimer Feld 227, 69120 Heidelberg, Germany

[2] Integrated Centre for Applied Physics and Photonic Materials, Technical University of Dresden, Nöthnitzer Str. 61, 01187 Dresden, Germany

[3] Center for Advancing Electronics Dresden (cfaed), Helmholtzstraße 18, 01069 Dresden, Germany

Corresponding Author

*Yana Vaynzof, e-mail: yana.vaynzof@tu-dresden.de

Supplementary Note 1: Calculation of the Fast and Slow Antisolvent Extrusion Rate

To calculate the antisolvent extrusion rate, five simulated antisolvent applications for both fast and slow were recorded on video using a dark liquid for contrast. Each video was then broken up into individual frames, wherein the frame at which the application started and finished was noted. Using the known frame rate, the duration for each was calculated and the average found. Dividing the volume (200 μL) by this average duration yielded the average rate for fast and slow application.

Supplementary Note 2: Antisolvent Characteristics

Supplementary Table 1: Physiochemical properties of all 14 solvents. The solvents are abbreviated as follows: EtOH is ethanol, IPA is isopropanol, BuOH is butyl alcohol, EA is ethyl acetate, CF is chloroform, CB is chlorobenzene, BA is butyl acetate, DCB is 1,2-dichlorobenzene, Ani is anisole, TFT is trifluorotoluene, DEE is diethyl ether, Xyl is m-xylene, and Tol is toluene, Mesit is mesitylene.

Type	Solvent	Density [g/ml]	Boiling point [°C]	Dipole moment [D]
**I**	EtOH	0.79	78	1.69
	IPA	0.79	83	1.66
	BuOH	0.81	118	1.66
**II**	EA	0.90	77	1.78
	CF	1.49	61	1.15
	CB	1.11	131	1.69
	BA	0.88	126	1.87
	DCB	1.30	180	2.50
	Ani	1.00	154	2.30
	TFT	1.19	103	2.86
**III**	DEE	0.71	35	1.15
	Xyl	0.86	139	0.33-0.37
	Tol	0.87	111	0.36
	Mesit	0.86	164.7	0.047

Supplementary Note 3: Sample Solar Cell Characteristics

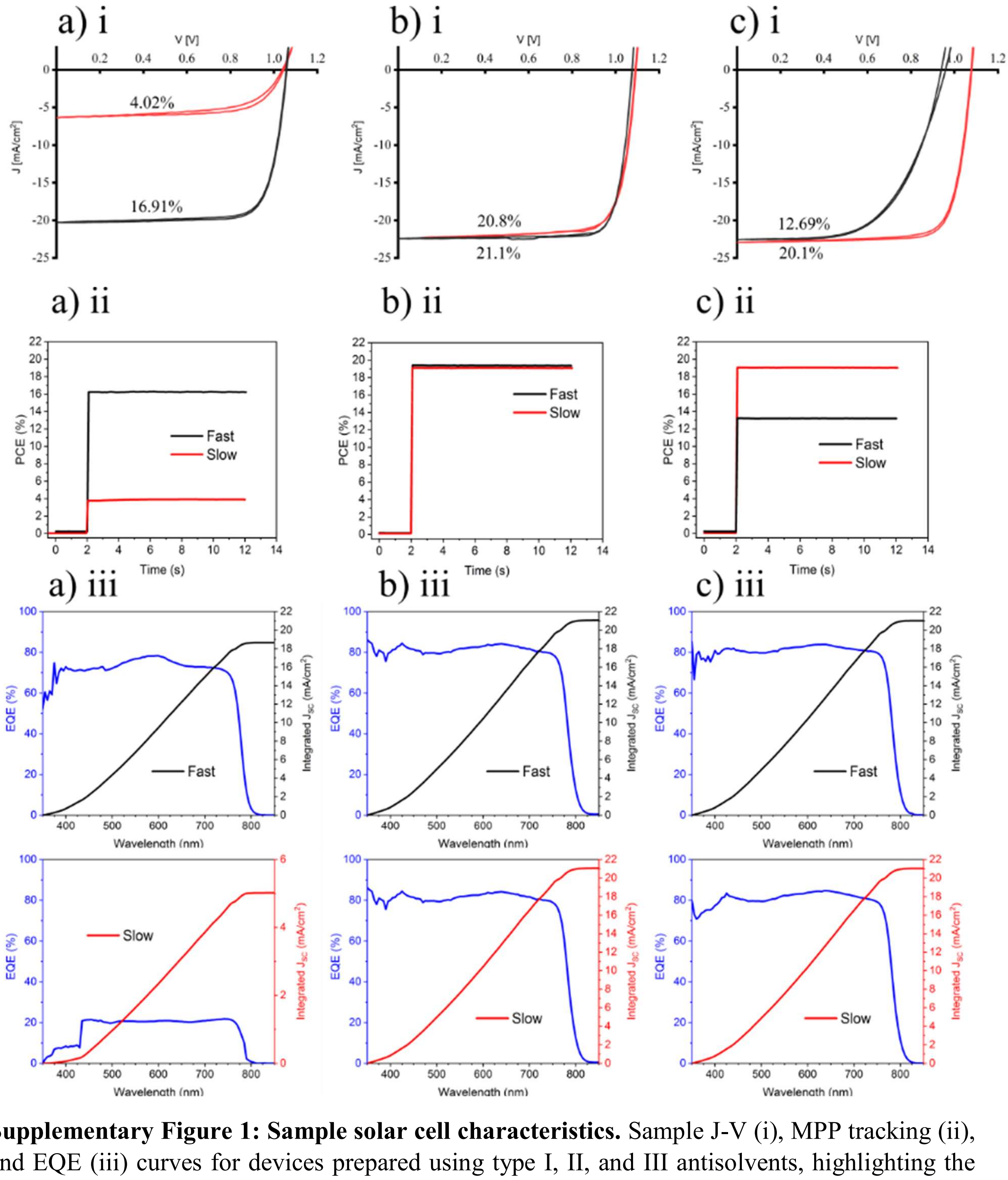

Supplementary Figure 1: Sample solar cell characteristics. Sample J-V (i), MPP tracking (ii), and EQE (iii) curves for devices prepared using type I, II, and III antisolvents, highlighting the differences between fast and slow antisolvent application. a) ethanol, b) anisole, c) toluene. Red indicates slow and black indicates fast.

Supplementary Note 4: Boundary Determination of Slow and Fast Antisolvent Application

a)

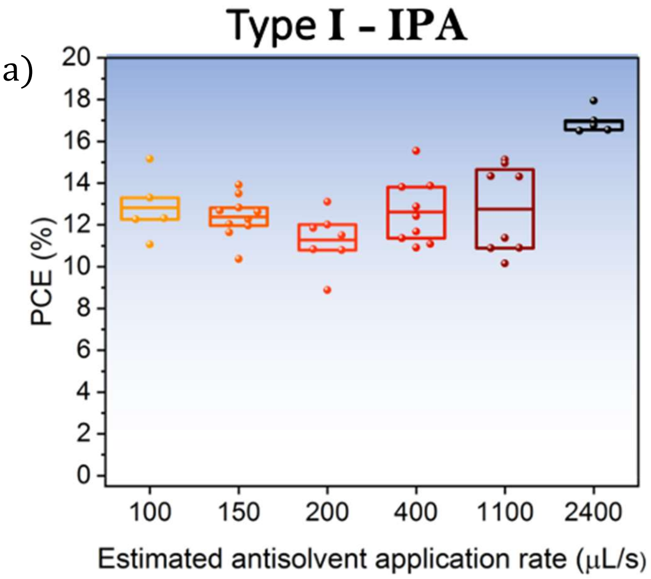

b)

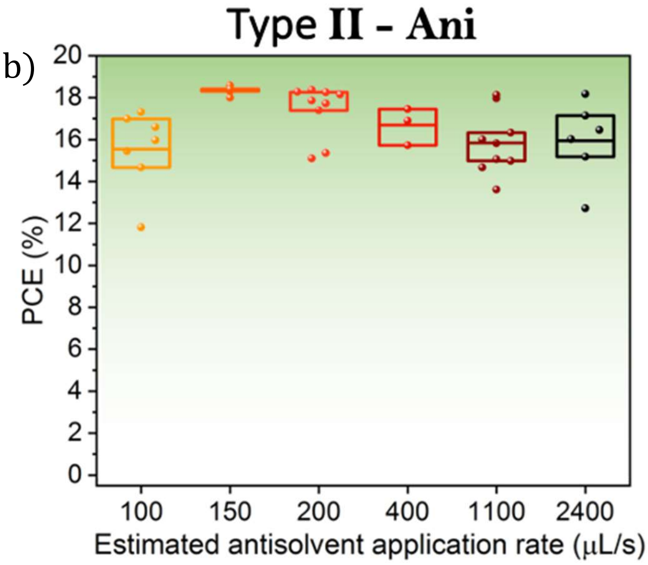

c)

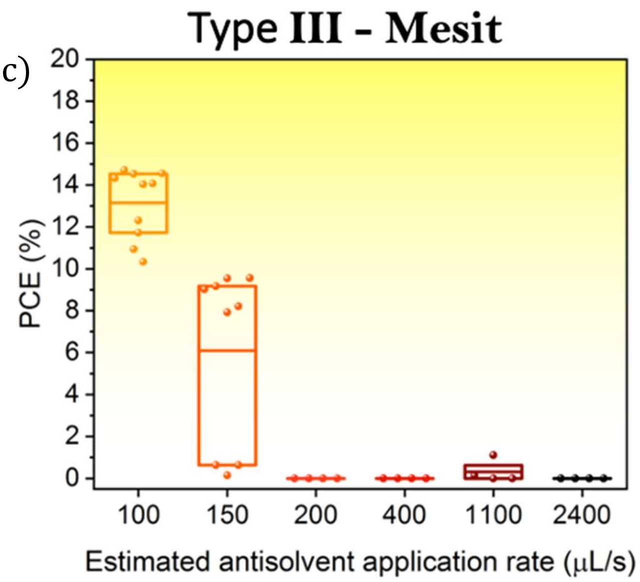

Supplementary Figure 2: Boundary Determination of Slow and Fast Antisolvent Application. Photovoltaic performance for a) type I (isopropyl alcohol), b) II (anisole), and c) III (mesitylene) antisolvents as a function of gradually changing application rate, to find the rate between fast and slow application at which the performance transitions. The transition rate for isopropyl alcohol appears at approximately 1100-1500 μL/s, and at roughly 100-150 μL/s for mesitylene. As a type II antisolvent, anisole possesses equal performance at all application rates. The center line denotes mean value, box limits are upper and lower quartiles.

Supplementary Note 5: XPS Measurements

Samples for X-ray photoemission spectroscopy (XPS) measurements were prepared as described above on PFN-Br/PTAA coated ITO substrates and then tranferred to the ultrahigh vacuum chamber of the XPS system (Thermo Scientific ESCALAB 250Xi). XPS measurements were performed using a XR6 monochromated Al Kα source (hν = 1486.6 eV) and a pass energy of 20 eV.

The iodine to lead atomic ratio (I/Pb) is calculated by dividing the atomic percentage of iodine by that of lead as obtained from the collected Pb*4f* and I*3d* spectra. The bromide to lead (Br/Pb), caesium to lead (Cs/Pb), FA to lead (FA/Pb) and MA to lead (MA/Pb) atomic ratios are obtaied in a similar way. The N*1s* spectrum of the triple cation perovskite exhibts two peaks at around 400.6 eV and 402.9 eV, representing the formamidinium (FA) and methylammonium (MA), respectively (Supplementary Figure 3a). The atomic percentages of the FA and MA are quantified by fitting the corresponding N1s peaks.

Note that the large variation in the MA/Pb among different acquisition spots for each single sample (Supplementary Figure 3b) is the result of the fitting, because the N*1s* peak assigned to MA is low and noisy.

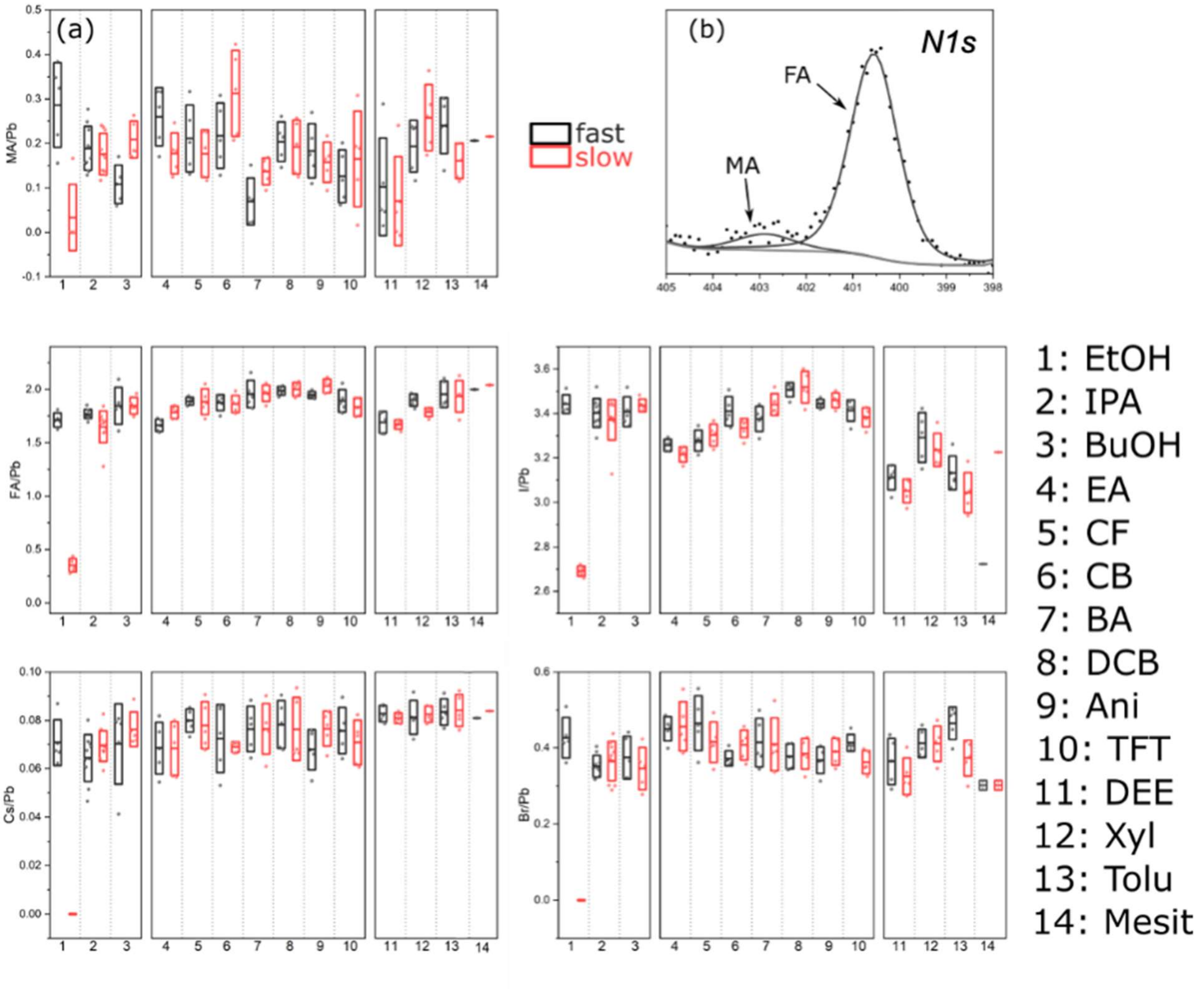

Supplementary Figure 3: **XPS characterization of fast vs. slow devices.** a) example N*1s* peaks, showing the signal separation of nitrogen peaks from MA and FA. b) Atomic ratios between lead and the other constituents (MA, FA, I, Br, Cs) of triple cation perovskite. The center line denotes mean value, box limits are upper and lower quartiles.

Supplementary Note 6: SEM and 2D-XRD for Type II Antisolvents

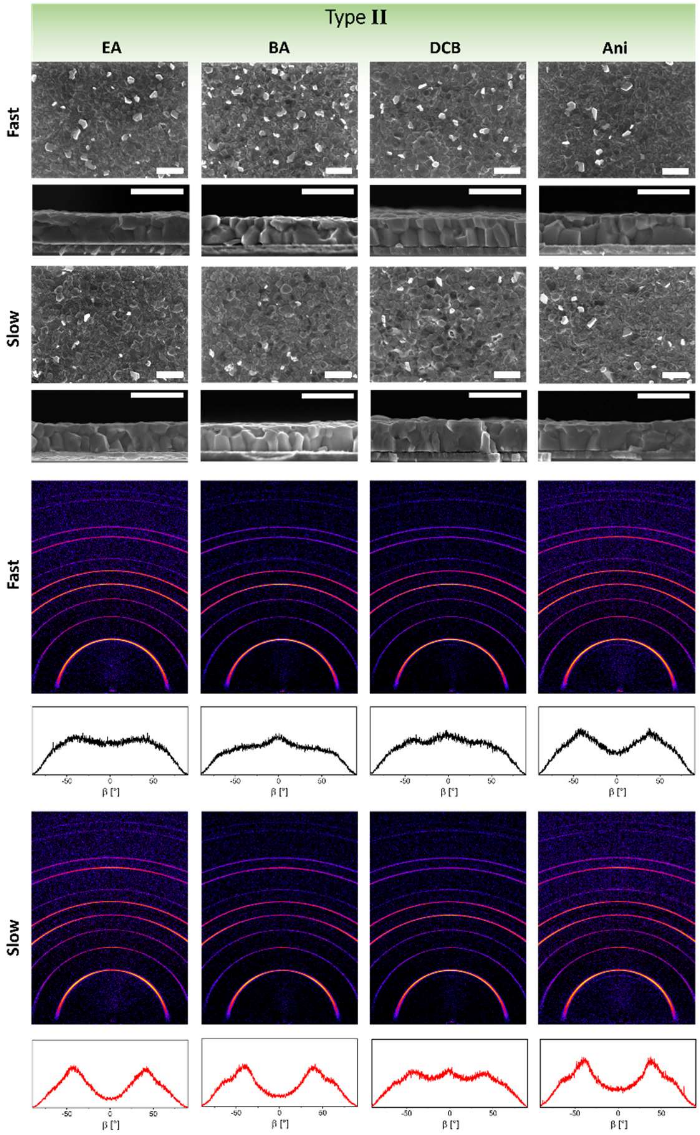

Supplementary Figure 4: **SEM and 2D-XRD for type II antisolvents.** Top: surface and cross-sectional scanning electron microscopy images of perovskite films formed from selected type II antisolvents ethyl acetate (EA), butyl acetate (BA), 1,2-dicholorobenzene (DCB), and anisole (Ani). Scale bar is 1 µm. Bottom: 2D XRD maps and corresponding ß integration of the (100) reflection to visualize changes in grain orientation.

Supplementary Note 7: Optical Images of Type II and III Antisolvent Treatments

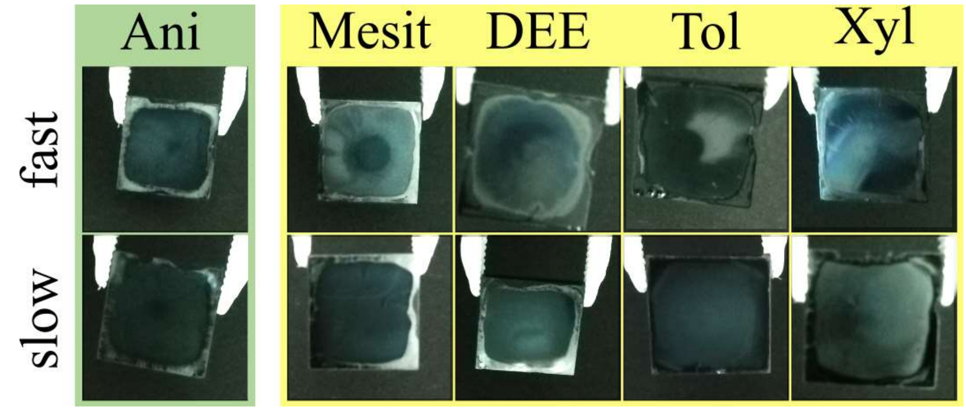

Supplementary Figure 5: Optical Images of Type II and III Antisolvent Treatments
Photographs of films fabricated using type III antisolvents mesitylene (Mesit), dithyl ether (DEE), toluene (Tol), and xylene (Xyl), with the type II antisolvent anisole (Ani) for reference, fabricated fast and slow. Note that the black/white contrast has been adjusted in order to more easily visualize differences between the films.

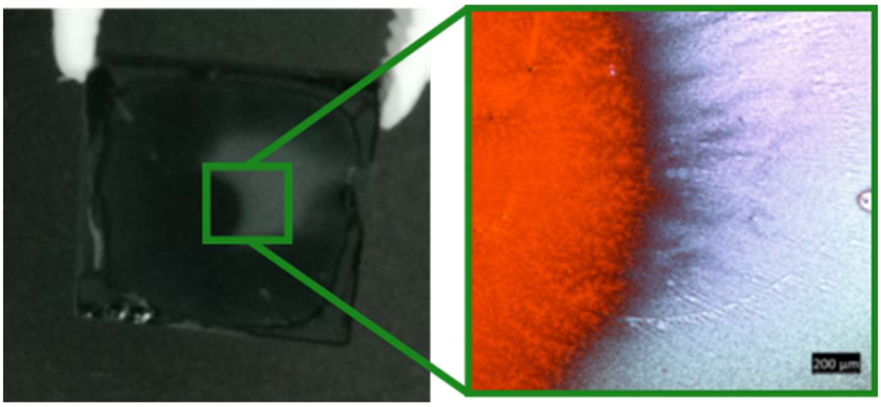

Supplementary Figure 6: Photograph of a perovskite film formed using toluene/fast, and the corresponding optical transmission microscopy image of the highlighted region. While the center circle is the high-quality perovskite seen via SEM, outside the circle is amorphous material.

Supplementary Note 8: SEM and 2D-XRD for Type III Antisolvent Mesitylene

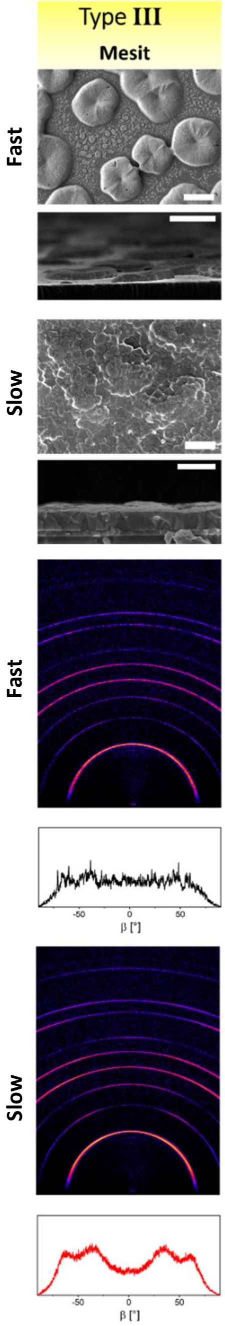

Supplementary Figure 7: **SEM and 2D-XRD for type III antisolvent mesitylene.** Top: surface and cross-sectional scanning electron microscopy images of perovskite films formed from type III antisolvent mesitylene (Mesit). Scale bar for fast antisolvent application is 10 µm and for slow antisolvent application is 1 µm. Bottom: 2D XRD maps and corresponding ß integration of the (100) reflection to visualize changes in grain orientation.

Supplementary Note 9: Solubility of FAI in DMF:DMSO and Antisolvents

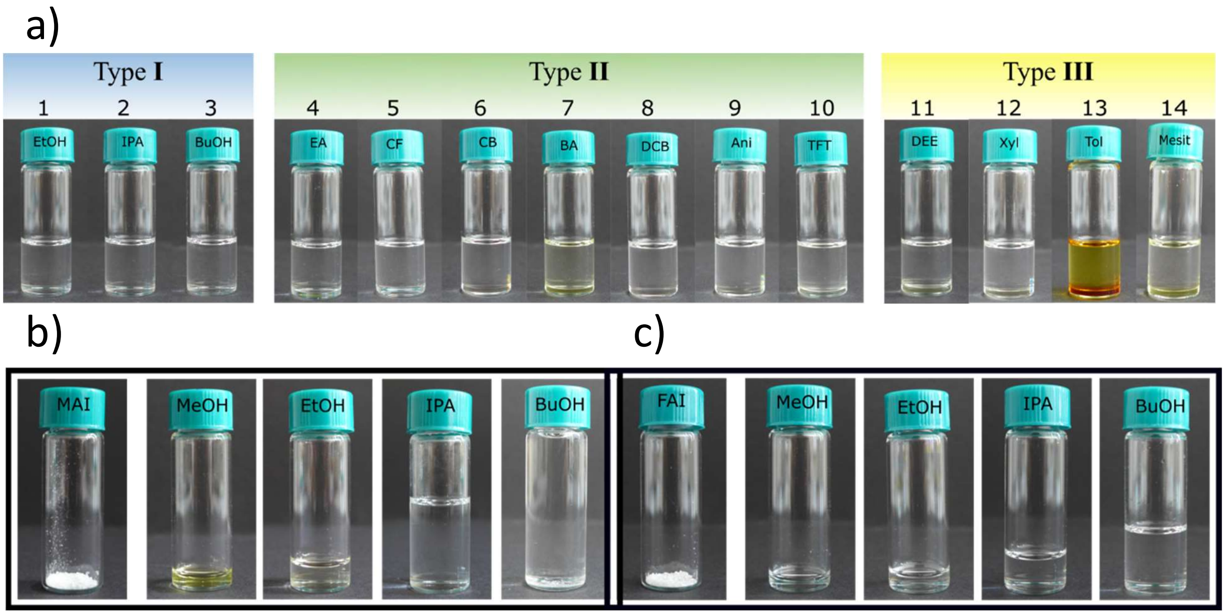

Supplementary Figure 8: Solubility of FAI in DMF:DMSO and antisolvents. a) Solubility of formamidinium iodide (FAI) in a solution of DMF:DMSO:antisolvent, meant to simulate the perovskite film intermediate phase during the antisolvent step of fabrication. b,c) Amount of solvent (alcohols) required to fully dissolve 100 mg of methylammonium iodide (MAI)* and FAI, respectively. *Note that only the MAI in the BuOH solution is not fully dissolved.

Supplementary Note 10: Optical Image of Film using Methanol as Antisolvent

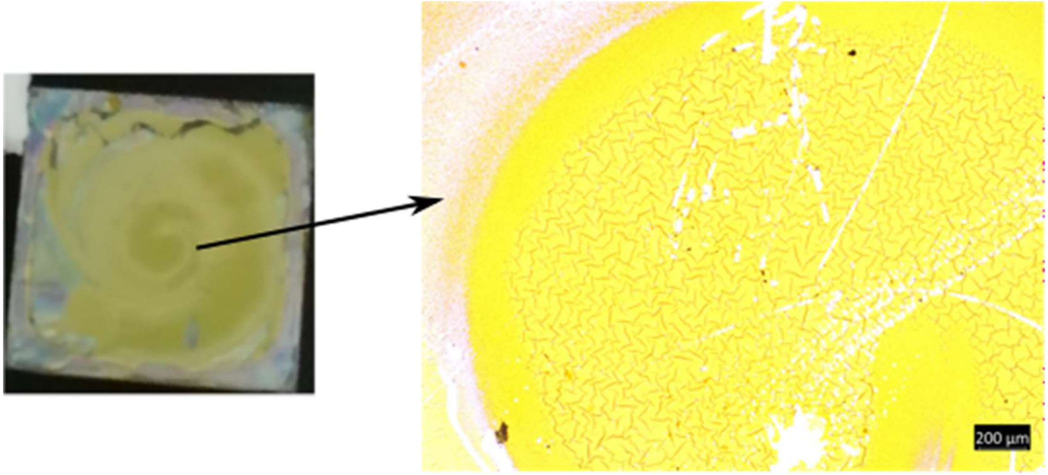

Supplementary Figure 9: Optical image of film using methanol as antisolvent. Film resulting from the use of methanol as antisolvent, leaving only residual PbI_2.

Supplementary Note 11: UV-Vis of Type III Antisolvents

A second phenomenon displayed by the type III solvents is the yellowish color change. UV-vis absorption measurements of the same 2 M MAI/FAI solutions in DMF:DMSO mixed with the type II antisolvents reveal absorption onsets and peaks consistent with iodide (I_2), as well as more reduced iodine species such as I_3^-, shown in Figure S10. These results indicate the possible degradation of the MAI/FAI molecules in solution. However, we note that this process is much slower than the duration during antisolvent application and is thus unlikely to affect the resulting film.

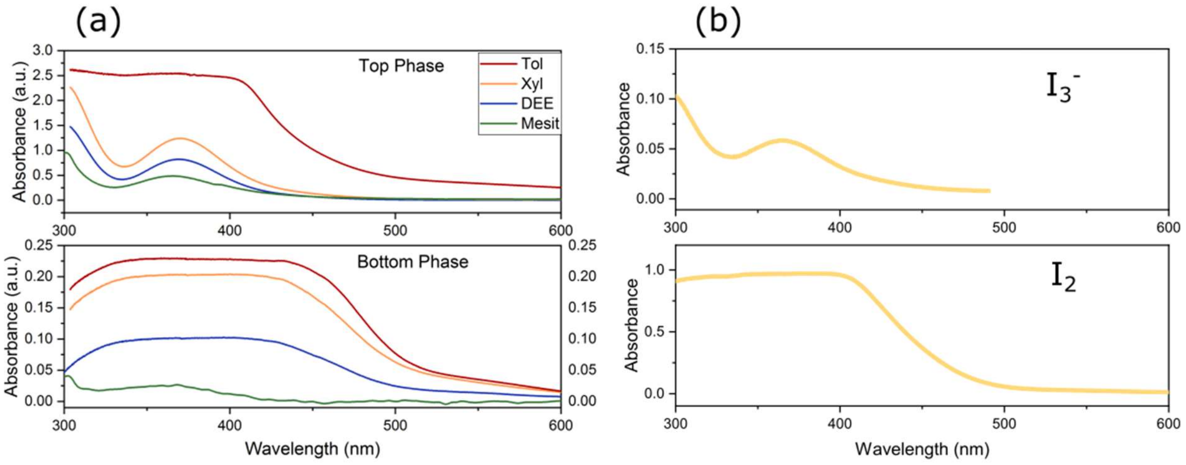

Supplementary Figure 10: **: UV-Vis absorbance of type III antisolvents.** a) UV-vis extinction spectra of 2 M MAI/FAI solutions, dissolved in 6:1 antisolvent:[DMF:DMSO], using the Type III antisolvents for the top and bottom separated liquid phases. b) Sample extinction spectra of I_2 and I_3^- for reference.[1]

Supplementary Note 12: Effect of Varying the Distance between the Pipette and Substrate

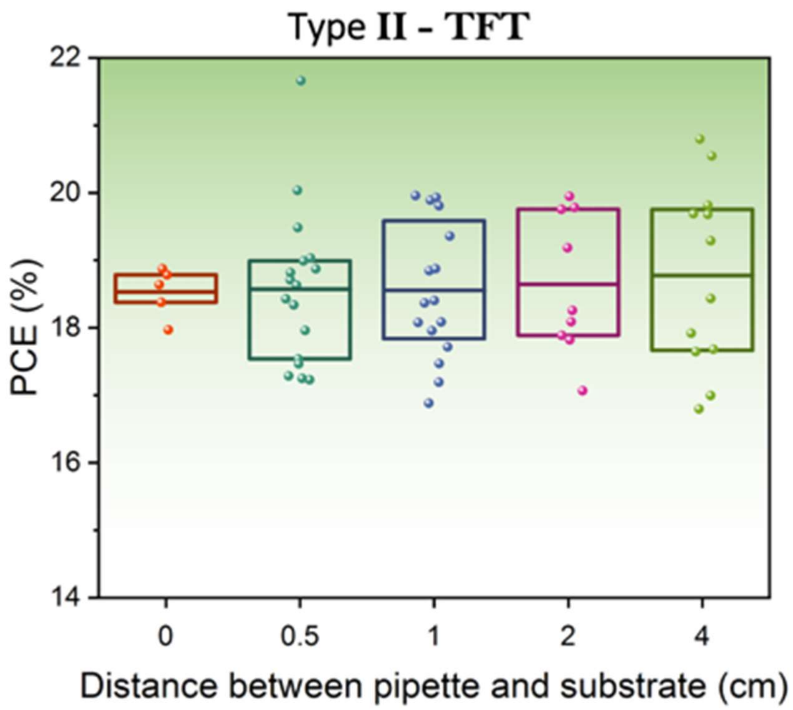

Supplementary Figure 11: **Effect of Varying the Distance between the Pipette and Substrate.** Photovoltaic performance as a function of the distance between the pipette and substrate when the antisolvent is applied. These devices fabricated using the type II antisolvent TFT with fast application. The center line denotes mean value, box limits are upper and lower quartiles.

Supplementary Note 13: Effect of Varying the Environment of Perovskite Layer Fabrication

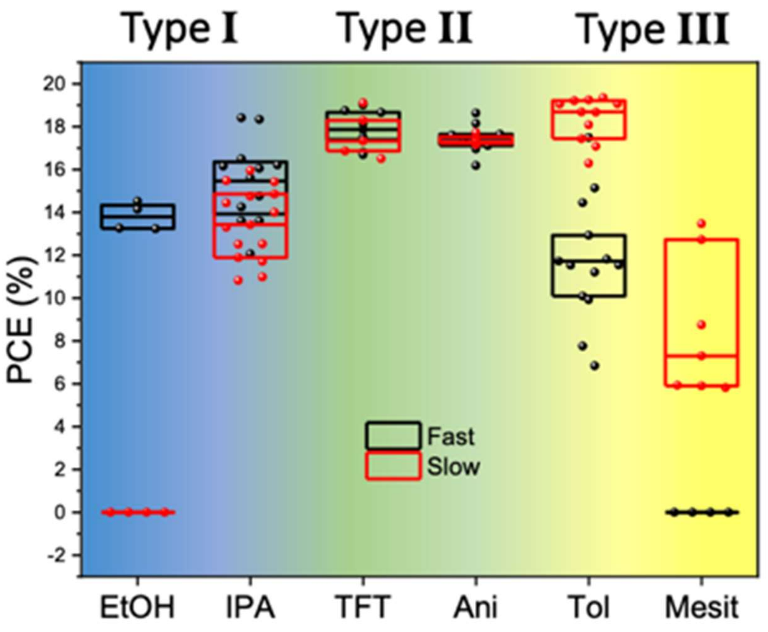

Supplementary Figure 12: Effect of Varying the Environment of Perovskite Layer Fabrication. Photovoltaic performance of devices resulting from a fast or slow antisolvent application fabricated in a N_2-filled glovebox instead of a dry air-filled glovebox, for type I (EtOH), II (Ani), and III (Tol) antisolvent. The center line denotes mean value, box limits are upper and lower quartiles.

Supplementary Note 14: Effect of Increasing the Antisolvent Temperature up to 50 °C

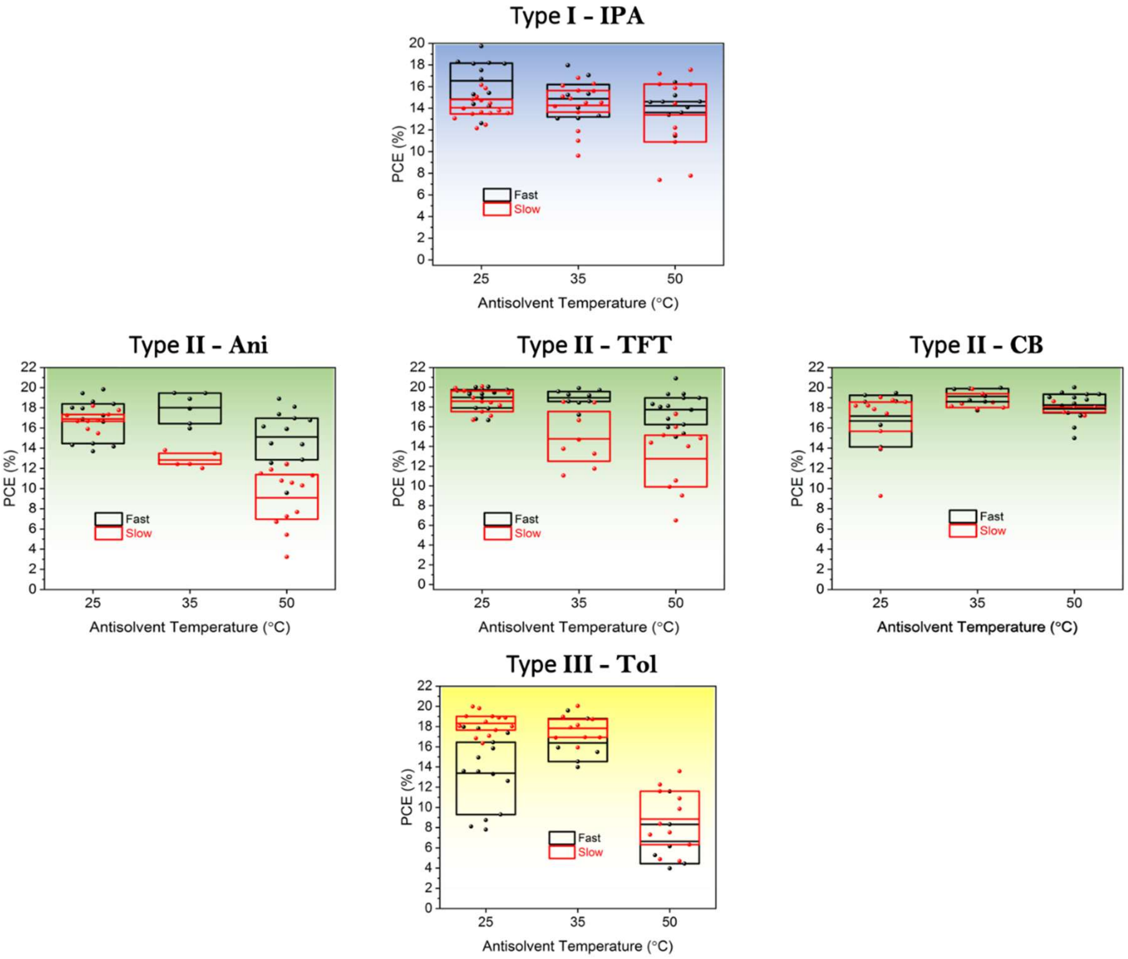

Supplementary Figure 13: Effect of increasing the antisolvent temperature. Photovoltaic performance of devices resulting from a fast or slow antisolvent application using antisolvent at room temperature (25), 35, and 50 °C. The center line denotes mean value, box limits are upper and lower quartiles.

Supplementary Note 15: Effect of Varying the Antisolvent Volume

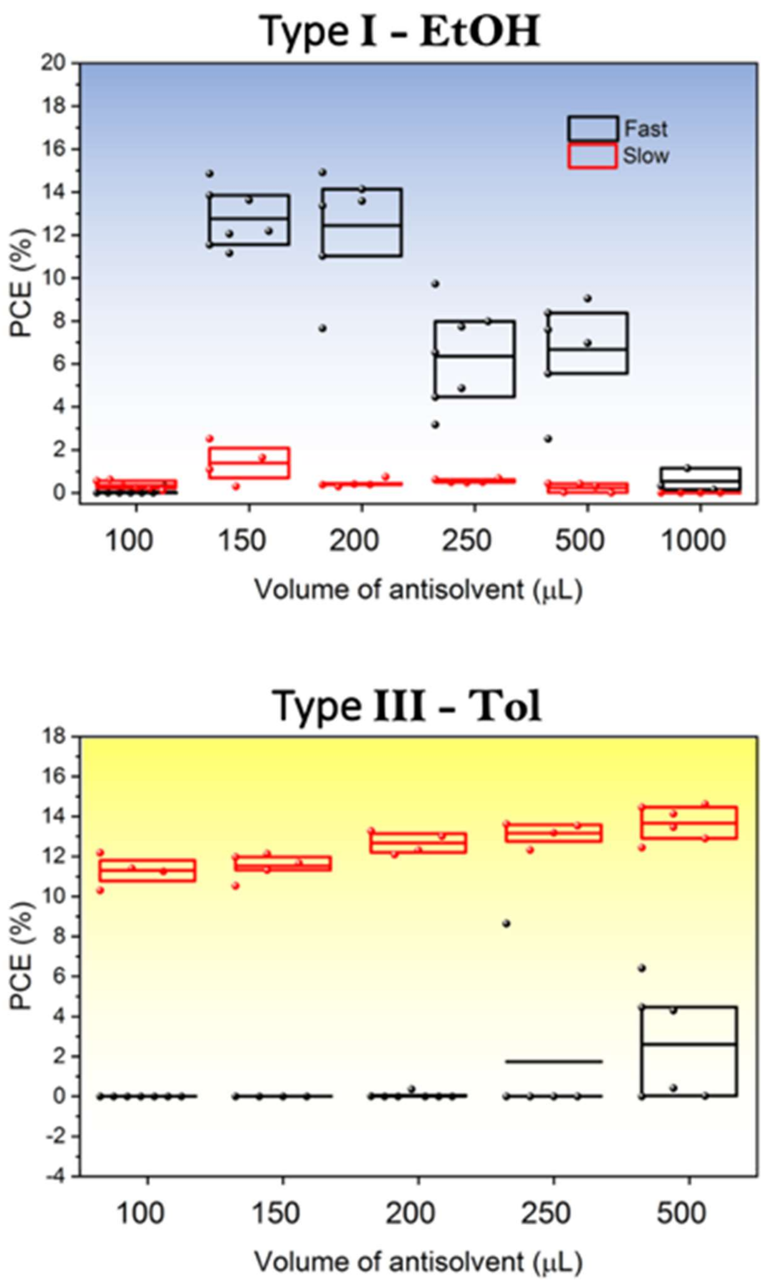

Supplementary Figure 14: Effect of Varying the Antisolvent Volume. Photovoltaic performance of devices resulting from a fast or slow antisolvent application fabricated using different volumes of antisolvent, for a type I (EtOH) and III (Tol) antisolvent. The center line denotes mean value, box limits are upper and lower quartiles.

Supplementary Note 16: Stability of Photovoltaic Devices Fabricated by Different Antisolvent Types

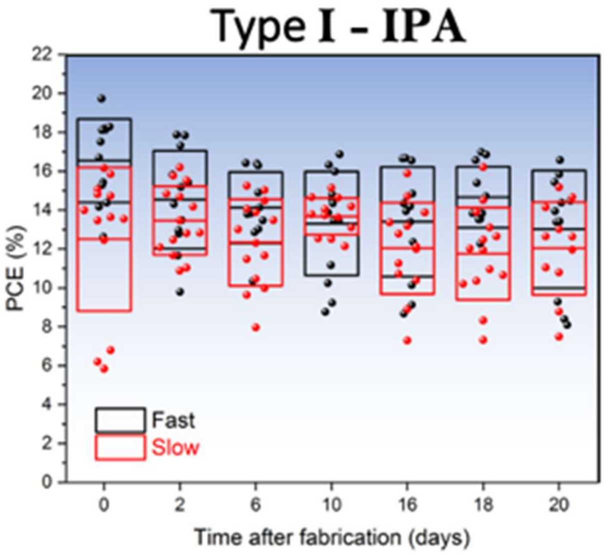

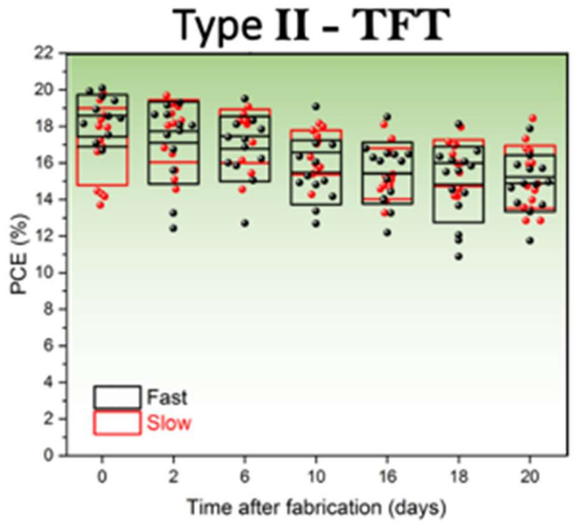

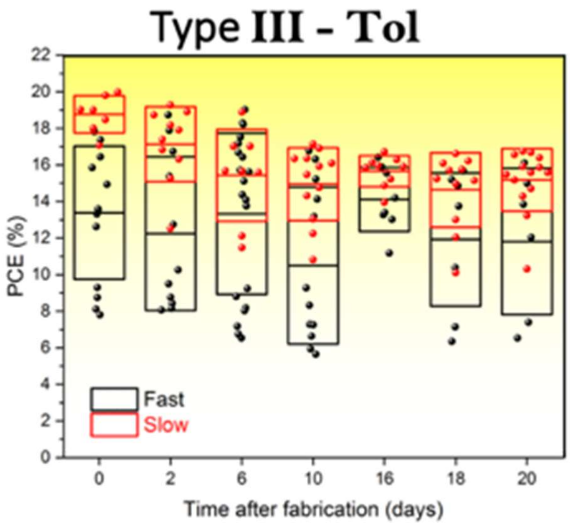

Supplementary Figure 15: Stability of photovoltaic devices fabricated by different antisolvent types. PV performance over 20 days for devices fabricated using a) IPA (type I), b) TFT (type II), and c) toluene (type III) antisolvents, for both fast and slow antisolvent application speeds. The center line denotes mean value, box limits are upper and lower quartiles. Devices were stored "on the shelf", i.e. in ambient air and in the dark.

Supplementary Note 17: Evolution of Perovskite Film Absorption over a Period of 4 Weeks

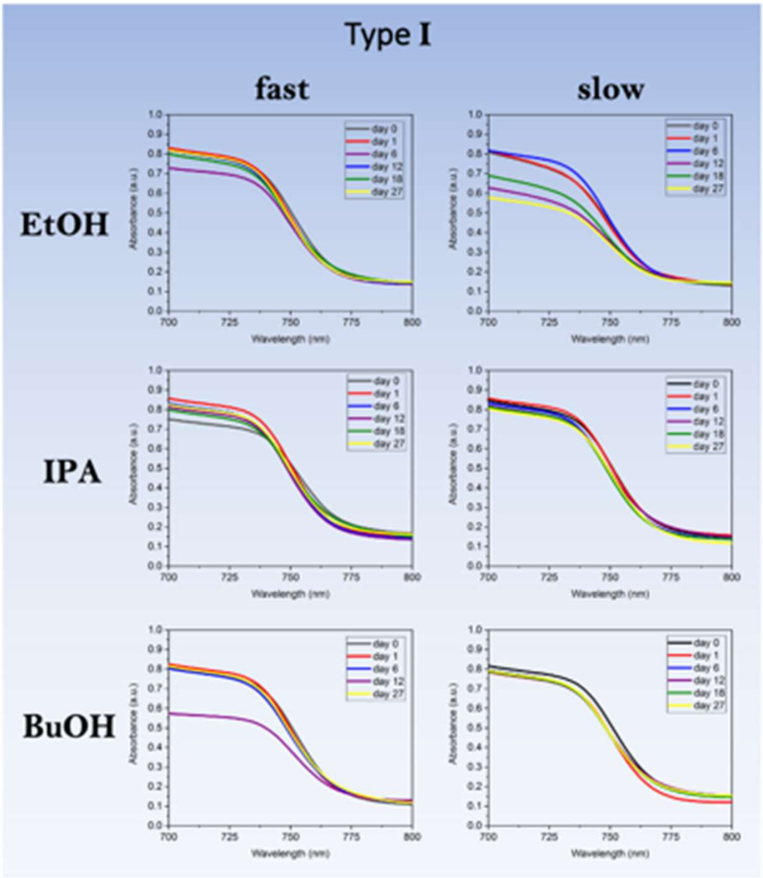

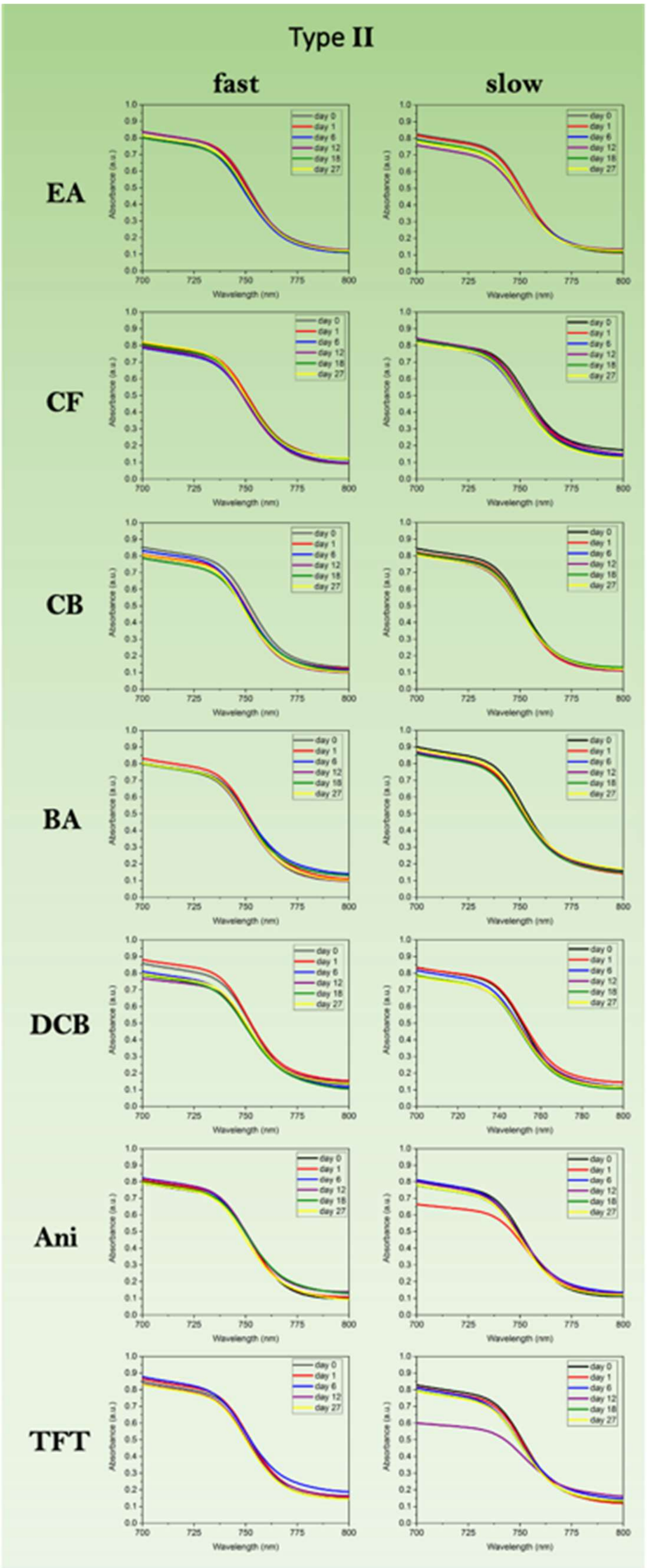
Type II
fast
slow
EA
CF
CB
BA
DCB
Ani
TFT
Absorbance (a.u.)
Wavelength (nm)
day 0
day 1
day 6
day 12
day 18
day 27

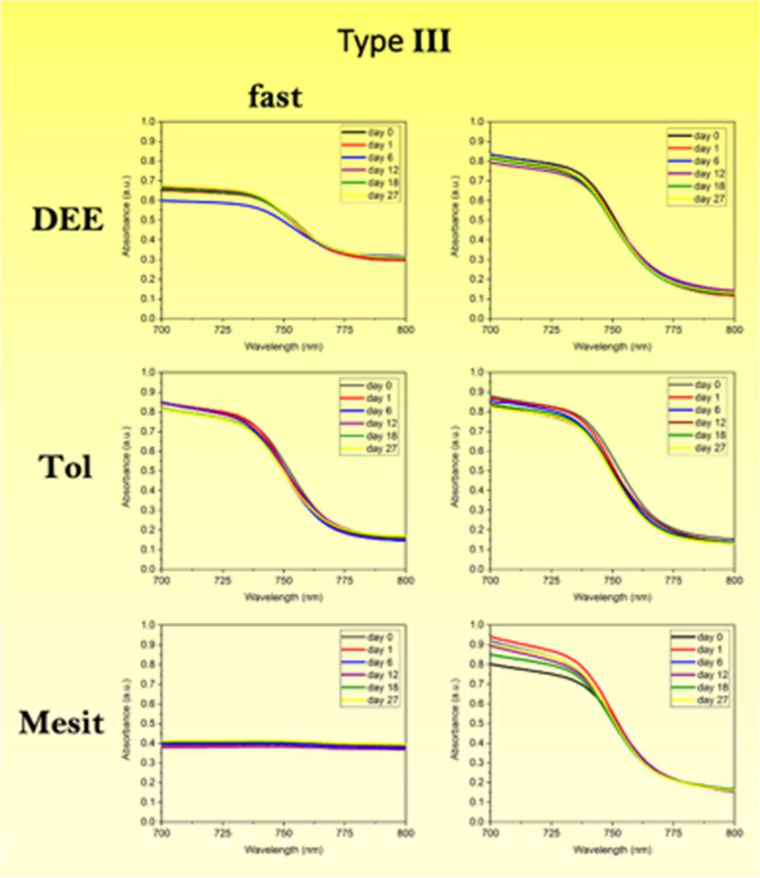

Supplementary Figure 16: Evolution of perovskite film absorption over a period of 4 weeks. Absorption onset of triple-cation perovskite films measured via ultraviolet-visible absorbance spectroscopy, deposited on ITO/PTAA/PFN-Br glass substrates, as a function of time over 4 weeks. Devices were stored "on the shelf", i.e. in ambient air and in the dark. Please note that certain outliers are present since different spots of the sample were measured each time.

Supplementary Note 18: Fabrication of Perovskite Films on Large Area Substrates

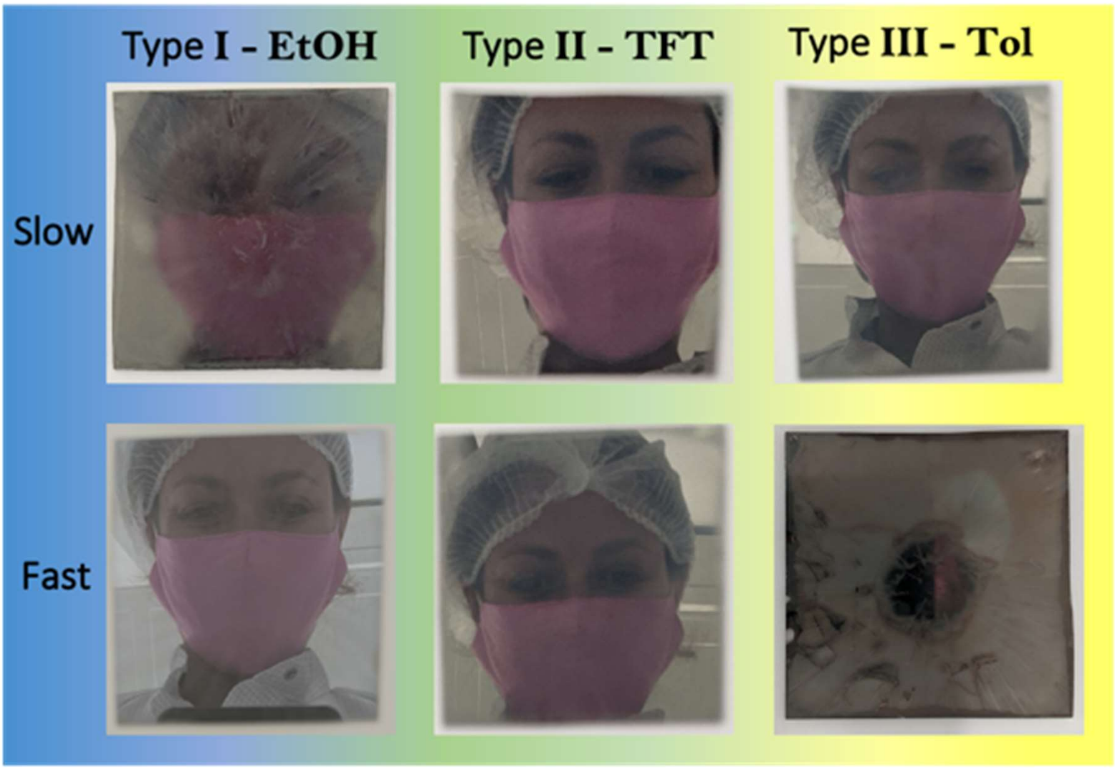

Supplementary Figure 17: Fabrication of Perovskite Films on Large Area Substrates. Photographs of large area samples (5x5 cm^2) fabricated using EtOH (Type I), TFT (Type II) and Tol (Type III) using a slow and fast application speed.

Supplementary Note 19: MA-Free Perovskite with Type I, II, and III Antisolvents

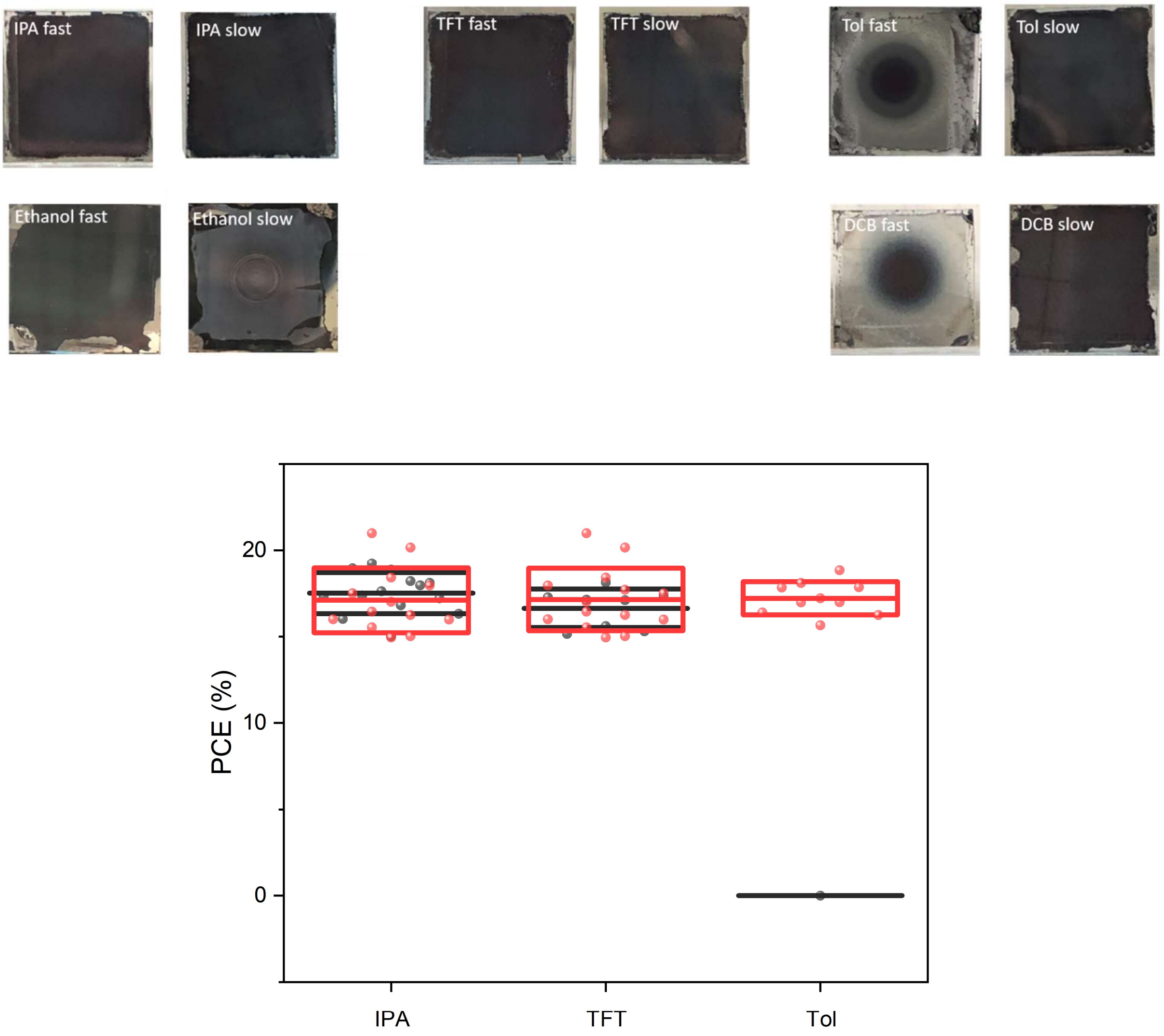

Supplementary Figure 18: MA-Free Perovskite with Type I, II, and III Antisolvents. Perovskite used was $Cs_{0.1}FA_{0.9}PbI_{2.9}Br_{0.1}$, with isopropyl alcohol (type I), trifluorotoluene (type II), and toluene (type III) antisolvents applied fast and slow. The center line denotes mean value, box limits are upper and lower quartiles.

Supplementary Note 20: Full PV Characteristics as a Function of Stoichiometry

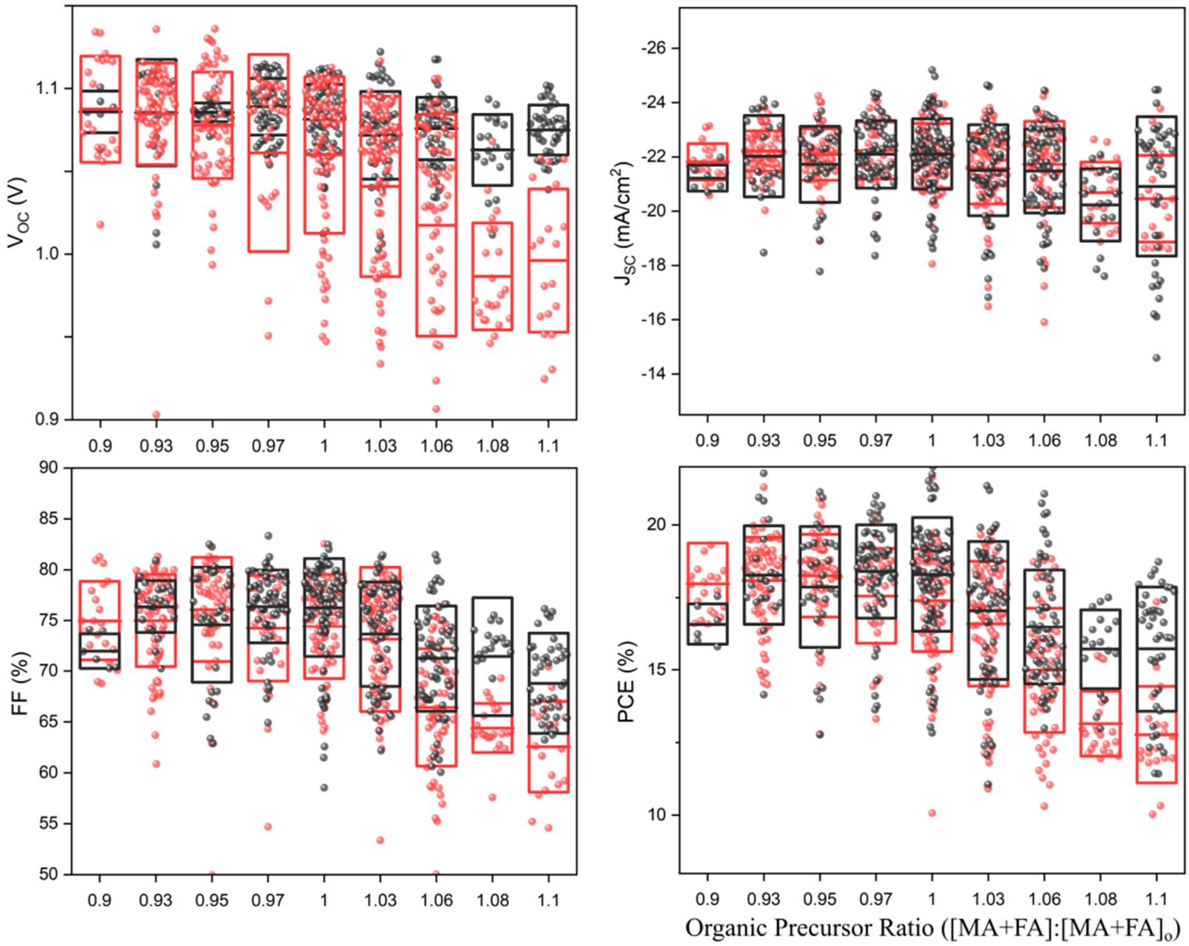

Supplementary Figure 19: Full PV characteristics as a function of stoichiometry. Figure 7 from the main text, with all of the data displayed instead of only the top 10 pixels. Note that the overall trends remain identical, but are simply more difficult to follow due to the large number of overlapping points. The center line denotes mean value, box limits are upper and lower quartiles.

Supplementary Note 21: 1D XRD Profiles of Perovskite Films Fabricated by all Antisolvents

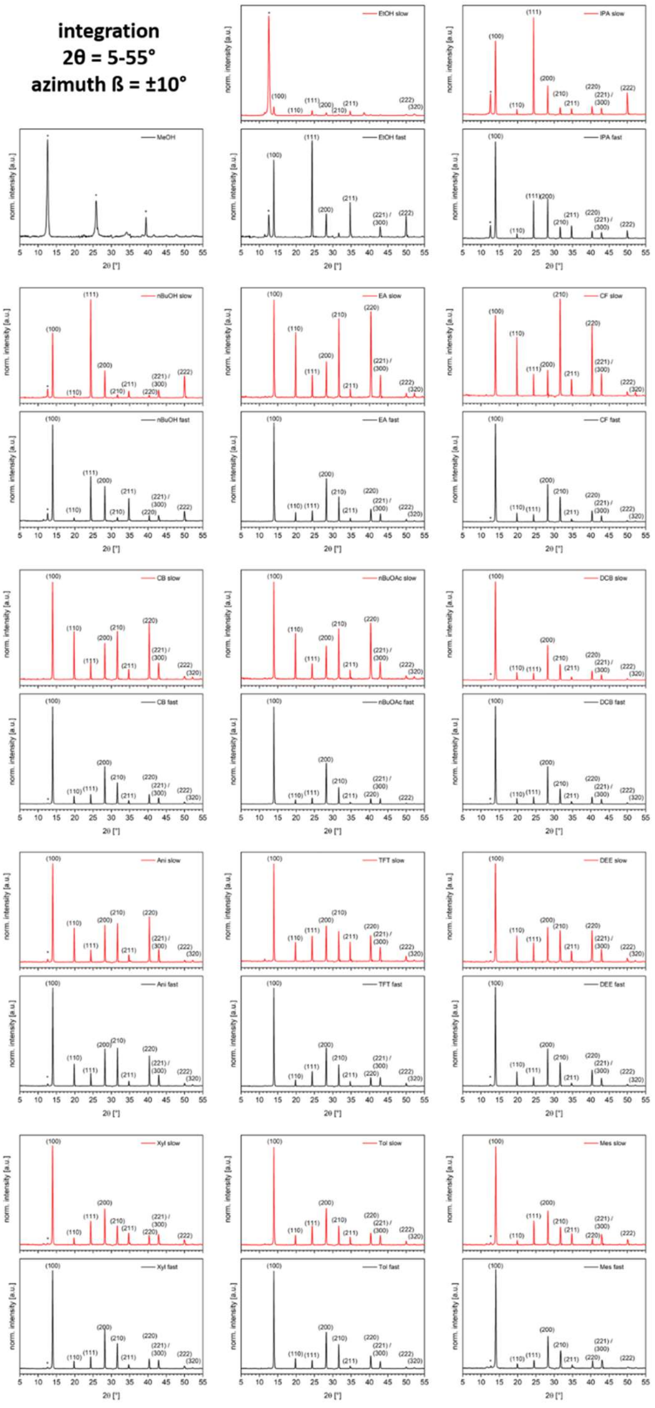

Supplementary Figure 20: 1D XRD Profiles of Perovskite Films Fabricated by all Antisolvents. 1D-diffraction profiles for all antisolvents. All samples exhibit diffraction pattern of cubic perovskite unit cell. Label * marks reflections attributed to PbI_2.

Supplementary Note 22: SEM Comparison of LABE and SEI Detectors

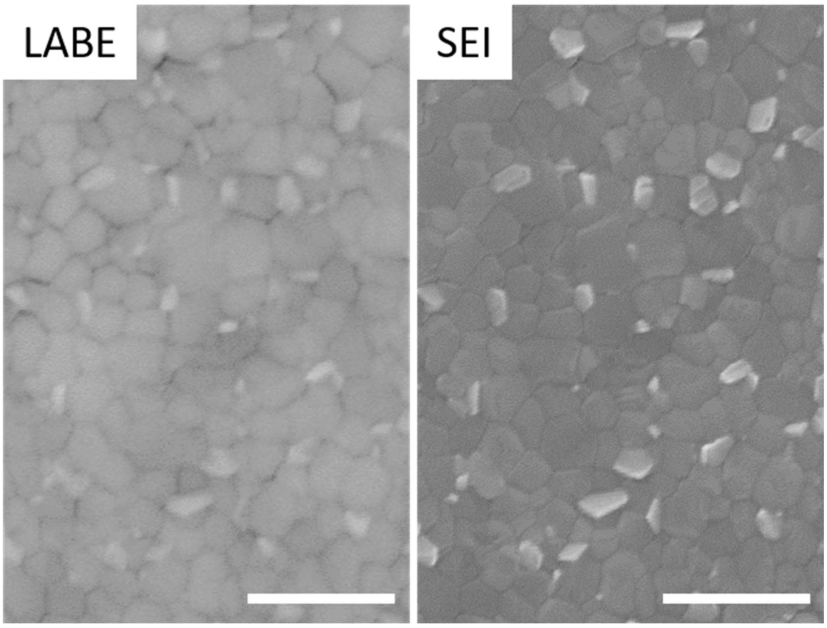

Supplementary Figure 21: SEM Comparison of LABE and SEI Detectors. SEM comparison of low-angle backscatter electron detector (LABE) and SEI detector of the same area of a perovskite film fabricated with fast antisolvent (solvent: ethyl acetate) treatment. Bright areas correspond to a material of higher density, in this case PbI_2.
