# Supplementary material for: A general approach to high-efficiency perovskite solar cells by any antisolvent
Source: Nat Commun. 2021 Mar 25;12:1878. doi: 10.1038/s41467-021-22049-8 (PMC7994557; doi:10.1038/s41467-021-22049-8)
Supplement: Supplementary file 2 — Solar Cells Reporting Summary [file 41467_2021_22049_MOESM2_ESM.pdf]

nature research

Corresponding author(s): Yana Vaynzof

# Solar Cells Reporting Summary

Nature Research wishes to improve the reproducibility of the work that we publish. This form is intended for publication with all accepted papers reporting the characterization of photovoltaic devices and provides structure for consistency and transparency in reporting. Some list items might not apply to an individual manuscript, but all fields must be completed for clarity.

For further information on Nature Research policies, including our data availability policy, see Authors & Referees.

## Experimental design

**Please check: are the following details reported in the manuscript?**

1. Dimensions

| Item | Yes | No | Details |
|---|---|---|---|
| Area of the tested solar cells | ☒ | ☐ | Methods section |
| Method used to determine the device area | ☐ | ☒ | Device area is permanently set by shadow mask during thermal evaporation. |

2. Current-voltage characterization

| Item | Yes | No | Details |
|---|---|---|---|
| Current density-voltage (J-V) plots in both forward and backward direction | ☒ | ☐ | All J-V curves |
| Voltage scan conditions<br>*For instance: scan direction, speed, dwell times* | ☒ | ☐ | Methods section |
| Test environment<br>*For instance: characterization temperature, in air or in glove box* | ☒ | ☐ | Methods section |
| Protocol for preconditioning of the device before its characterization | ☒ | ☐ | Methods section |
| Stability of the J-V characteristic<br>*Verified with time evolution of the maximum power point or with the photocurrent at maximum power point; see ref. 7 for details.* | ☒ | ☐ | Methods section, Supplementary note 3 |

3. Hysteresis or any other unusual behaviour

| Item | Yes | No | Details |
|---|---|---|---|
| Description of the unusual behaviour observed during the characterization | ☐ | ☒ | Negligible hysteresis was observed |
| Related experimental data | ☐ | ☒ | See above |

4. Efficiency

| Item | Yes | No | Details |
|---|---|---|---|
| External quantum efficiency (EQE) or incident photons to current efficiency (IPCE) | ☒ | ☐ | Supplementary note 3 |
| A comparison between the integrated response under the standard reference spectrum and the response measure under the simulator | ☒ | ☐ | Methods section |
| For tandem solar cells, the bias illumination and bias voltage used for each subcell | ☐ | ☒ | N/A |

5. Calibration

| Item | Yes | No | Details |
|---|---|---|---|
| Light source and reference cell or sensor used for the characterization | ☒ | ☐ | Methods section |
| Confirmation that the reference cell was calibrated and certified | ☒ | ☐ | Methods section |

Calculation of spectral mismatch between the reference cell and the devices under test

☒ Yes ☐ No — Methods section

6. Mask/aperture

Size of the mask/aperture used during testing

☐ Yes ☒ No — N/A

Variation of the measured short-circuit current density with the mask/aperture area

☐ Yes ☒ No — N/A

7. Performance certification

Identity of the independent certification laboratory that confirmed the photovoltaic performance

☐ Yes ☒ No — N/A

A copy of any certificate(s)
*Provide in Supplementary Information*

☐ Yes ☒ No — N/A

8. Statistics

Number of solar cells tested

☒ Yes ☐ No — Manuscript text

Statistical analysis of the device performance

☐ Yes ☒ No — N/A

9. Long-term stability analysis

Type of analysis, bias conditions and environmental conditions
*For instance: illumination type, temperature, atmosphere humidity, encapsulation method, preconditioning temperature*

☐ Yes ☒ No — N/A
